# Identifying subpopulations in multicellular systems by quantitative chemical imaging using label-free hyperspectral CARS microscopy†

**DOI:** 10.1039/d0an02381g

**Published:** 2021-02-12

**Authors:** Iestyn Pope, Francesco Masia, Kenneth Ewan, Ana Jimenez-Pascual, Trevor C. Dale, Florian A. Siebzehnrubl, Paola Borri, Wolfgang Langbein

**Affiliations:** Cardiff University, School of Biosciences Sir Martin Evans Building Museum Avenue Cardiff CF10 3AX UK borrip@cf.ac.uk; Cardiff University, School of Biosciences, European Cancer Stem Cell Research Institute Hadyn Ellis Building Maindy Rd Cardiff CF24 4HQ UK; Cardiff University, School of Physics & Astronomy The Parade Cardiff CF24 3AA UK langbeinww@cf.ac.uk

## Abstract

Quantitative hyperspectral coherent Raman scattering microscopy merges imaging with spectroscopy and utilises quantitative data analysis algorithms to extract physically meaningful chemical components, spectrally and spatially-resolved, with sub-cellular resolution. This label-free non-invasive method has the potential to significantly advance our understanding of the complexity of living multicellular systems. Here, we have applied an in-house developed hyperspectral coherent anti-Stokes Raman scattering (CARS) microscope, combined with a quantitative data analysis pipeline, to imaging living mouse liver organoids as well as fixed mouse brain tissue sections xenografted with glioblastoma cells. We show that the method is capable of discriminating different cellular sub-populations, on the basis of their chemical content which is obtained from an unsupervised analysis, *i.e.* without prior knowledge. Specifically, in the organoids, we identify sub-populations of cells at different phases in the cell cycle, while in the brain tissue, we distinguish normal tissue from cancer cells, and, notably, tumours derived from transplanted cancer stem cells *versus* non-stem glioblastoma cells. The ability of the method to identify different sub-populations was validated by correlative fluorescence microscopy using fluorescent protein markers. These examples expand the application portfolio of quantitative chemical imaging by hyperspectral CARS microscopy to multicellular systems of significant biomedical relevance, pointing the way to new opportunities in non-invasive disease diagnostics.

## Abstract 2

Quantitative hyperspectral coherent Raman scattering microscopy merges imaging with spectroscopy and utilises quantitative data analysis algorithms to extract chemical components, spectrally and spatially-resolved, with sub-cellular resolution.

## Graphical abstract

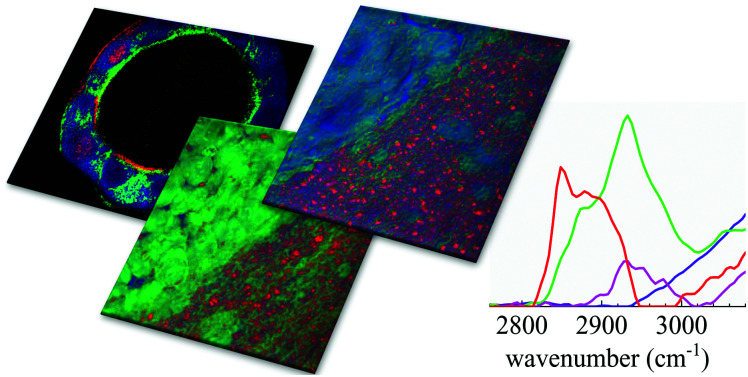

## 1. Introduction

It is becoming increasingly important in modern biomedical research to develop analytical tools capable of dissecting the complexity of multicellular systems, such as tissues and organs, without perturbing their natural state. Optical microscopy is still the only practical means to observe living specimens at sub-cellular spatial resolution in three dimensions and in real time. A major challenge of optical microscopy applied to cell biology is chemical specificity, since many biomolecules do not selectively absorb or emit light in the visible range.

Albeit some endogenous biomolecules do exhibit distinguishable autofluorescence emission, for example flavins and lipo-pigments,^1^ a widely utilized method to introduce chemical contrast is by means of exogenous fluorescent labels with high absorption cross-sections and quantum yields attached to biomolecules of interest. However, all organic fluorescent probes are prone to photobleaching, and associated phototoxicity, severely limiting long time-course observation in living specimens. Moreover, the process of labelling is often time-consuming, invasive, and can introduce staining artefacts. Over the past two decades, vibrational microscopy exploiting the phenomenon of Raman scattering has emerged as a powerful label-free, chemically-specific modality complementing fluorescence.^2^ Spontaneous Raman scattering cross-sections of biomolecules are notoriously small, with corresponding limitations in imaging speed and sensitivity. The development of coherent Raman scattering (CRS) microscopy^3^ is partially circumventing these limitations, owing to the constructive interference of Raman light scattered by identical bonds in the focal volume, coherently driven to vibrate in phase.

Many of the earlier demonstrations of CRS microscopy^4,5^ showed images acquired at a single vibrational resonance, often chosen at the CH_2_ symmetric stretch vibration around 2850 cm^−1^, which is abundant in lipids. However, it is well known from Raman micro-spectroscopy that the best chemical specificity is obtained when acquiring vibrational spectra spanning a relevant range of wavenumbers, at each spatial position. Such combination of imaging with spectroscopy in CRS, often called hyperspectral CRS microscopy, has seen various experimental implementations whereby spectra at each spatial point are acquired at once^6^ using a spectrometer with a camera, or sequentially, by tuning the excitation wavelength^7^ or *via* spectral focusing.^8^ Hyperspectral acquisition has significantly widened the application space of CRS microscopy, with recent reports showing the spatial distributions of not only lipids, but also proteins, water, and nucleic acid components inside cells^9–11^ and tissues,^6,7,12^ as well as the ability to distinguish different lipid types, such as saturated *versus* unsaturated fatty acids,^13,14^ cholesterol,^15^ and phospholipids.^16^ Building from these capabilities, the application of CRS microscopy to tissue analysis for disease diagnostics in histopathology has been gaining increasing attention.^17–19^ Notably, the photo-stability and reproducibility of CRS micro-spectroscopy makes it a quantitative technique, with the potential to overcome qualitative and subjective decision making processes in current medical practice, suffering from high degrees of variability.

The combination of hyperspectral CRS data acquisition with quantitative image analysis algorithms is an area which has received major interest in recent years. Broadly speaking there are two main ways in which hyperspectral data sets, consisting of CRS signals as a function of spatial and spectral coordinates, are visualised. Some groups report spatially-resolved CRS intensities, or retrieved Raman-like susceptibilities, at selected wavenumbers, the latter attributed to characteristic vibrational resonances thought to be specific of certain biomolecular species.^17^ Pseudocolor images are then formed by assigning colours to single-frequency spatial distributions and overlay them to highlight differences in spatial patterns, which in turn can serve as markers of *e.g.* sub-cellular structures (such as nuclei), or diseased *versus* normal cells in tissues.^6,12^ A more advanced quantitative approach consists in factorising hyperspectral data sets into the superposition of separate components having characteristic spectra, and visualising the spatially resolved map of the components. A few methods have been proposed in the latter approach, including principal component analysis^20^ and independent component analysis.^7^ However, these methods usually are not able to make an unsupervised decomposition into individual chemical species with absolute concentration determination, which is ultimately the most meaningful quantitative representation.

We have developed over the past years a hyperspectral coherent anti-Stokes Raman scattering (CARS) microscope and an unsupervised quantitative data-analysis algorithm which allows us to retrieve Raman-like spectra and spatially-resolved concentration maps of chemical components, in physically meaningful units, with no prior knowledge.^9–11,16,21^ The method has so far been applied to 2D cell cultures, and its capability to dissect more complex features in multicellular systems such as 3D cell cultures and tissues has not yet been showcased. In this work, we report the application of this method on two exemplar multicellular systems, namely mouse liver organoids and mouse brain tissues xenografted with glioblastoma (GBM) cells.

Organoids are 3D micro-physiological structures of 20–400 μm diameter formed from clusters of parenchymal cells that grow and self-organise within gels formed from matrix components. The careful definition of growth factor combinations has allowed organoids to be established from a range of normal tissues and from pathological samples, using both mouse models and cells extracted from humans/patients.^22^ Organoids are already the system of choice for the identification of therapies in conditions such as Cystic Fibrosis and are starting to emerge as systems for cancer drug discovery in personalised medicine, since they better recapitulate patient responses than 2D cancer cell lines.

Advances in cancer research over the last decade have highlighted molecular differences at the single cell level within the same cancer and have led to a greater recognition of the degree of heterogeneity within tumors. The brain cancer glioblastoma is one of the most heterogeneous types of cancer and its rapid progression has been linked to a subpopulation of cancer cells known as GBM cancer stem cells (GSCs).^23^ GSCs are capable of initiating and sustaining tumor growth and are more resistant to conventional therapies.^24,25^ GSCs also contribute to metabolic heterogeneity in brain cancer^26^ and it was previously shown that GSCs preferentially utilize lipid metabolism, while non-stem GBM cells (NGC) depend on glucose.^27^ Hence, profound biochemical differences exist between GBM cellular subsets. Most studies investigating tumor heterogeneity in GBM and other cancers rely on transcriptional profiling or other biochemical characterization of cancer cells which necessitate sample lysis and therefore cannot be performed on living cells. Live cell imaging studies shown so far critically depend on labels (fluorescent or otherwise) to enable detection of chemical changes within cells, which is limited by the chemical sensitivity and read-out of the reporter.

Here, we have investigated whether our quantitative label-free CARS microscopy method can be used to discriminate different cellular subpopulations within organoids and brain tissues, based on their endogenous chemical content. Specifically, we address if cells can be distinguished while at different phases during the cell cycle in liver organoids, and if GSCs *versus* NGCs can be separated in brain tissues.

## 2. Materials and methods

### 2.1. Sample preparation – Fucci2aR liver bile duct organoids

#### 2.1.1. Mice

The Fucci2aR construct, a fluorescence ubiquitination-based cell cycle indicator, is used to identify where a cell is within its cell cycle. Mice containing the bicistronic R26–Fucci2aR allele^28^ in the Rosa26 locus express the mCherry-hCdt and the mVenus-hGem fusion proteins upon expression of Cre protein. The mCherry-hCdt proteins and the mVenus-hGem proteins are only stable in the G1 and S/G2/M phases of the cell cycle, respectively. To be able to induce expression of the Fucci2aR construct in liver cells, R26–Fucci2aR allele mice in C57Bl/6-FvB background were crossed with C57Bl/6-FvB background mice bearing the Ahcre allele^29^ that strongly expresses the Cre protein in liver cells after administration of β-naphthoflavone. Mice were genotyped by PCR for presence of the R26–Fucci2aR and Ahcre alleles as described.^28,29^ Mice were held at the Beatson Institute, Glasgow on a 12 h light/dark cycle with free access to standard chow and water. To induce expression of the Fucci2aR construct, mice were injected intraperitoneally with 80 mg kg^−1^ β-naphthoflavone daily for 4 days using an 8 mg ml^−1^ β-naphthoflavone (Sigma, N3633) solution in corn oil (Sigma, C8267).

#### 2.1.2. Organoid culture

Mice were euthanised on the day after the final β-naphthoflavone injection and liver tissue dissected out. Liver bile duct organoid cultures were derived from diced liver fragments and were cultured and passaged as described using the Expansion Medium formulation.^30^ To optimise fluorescence strength, liver bile duct cultures were trypsinised to single cells with TrypLE (Thermo-Fisher, Invitrogen) and gated on a BD FACSDiva 8.0.1 (Becton Dickson) for the strongest mCherry-hCdt fluorescence which were used in subsequent experiments. To set up cultures for imaging, a piece of silicone gasket containing four holes of 3 mm diameter was cut from a 1 mm thick CultureWell gasket (Grace Bio-Labs, SKU: 103250), ethanol-sterilised and put in the middle of a low profile, uncoated, sterile 35 mm diameter Dish (Ibidi, IB-80131). After passage of liver bile duct organoids, 8 μl of Growth Factor Reduced Matrigel (Corning, 354230) containing liver bile duct organoid fragments was pipetted into each well in the gasket and allowed to set (at 37 °C for 5 min). The cultures were then covered in medium. Lids were replaced with differential interference contrast (DIC) glass insert lids (Ibidi, IB-80050) for DIC imaging. Cultures were imaged 2–3 days after seeding.

### 2.2. Sample preparation – brain tissues with GBM

Human glioblastoma cell line L0 was a kind gift from Prof. Brent Reynolds (University of Florida) and was cultured as described previously.^31^ Briefly, cells were lentivirally transduced to express the green fluorescent protein eGFP and maintained as sphere cultures in N2 medium containing 20 ng ml^−1^ EGF. For orthotopic implantation of GSC and NGC populations, glioblastoma cells were dissociated, and single cell suspensions immunostained using a mouse monoclonal antibody against FGFR1 (Thermo Fischer, 1:50 dilution) and appropriate secondary antibody (Thermo Fischer, 1:500), and purified by FACS.^31^ Orthotopic xenografts of FGFR1 + (GSC) and FGFR1-(NGC) populations (10 000 cells per animal each) were performed in 4–6 weeks old female SCID mice.^31^ Animal care and handling, and all procedures were performed in accordance with FELASA and institutional guidelines of Cardiff University and approved by the Animal Welfare Ethical Review Body of Cardiff University and the UK home office (PPL30/3331). Mice were maintained under Isoflurane anesthesia during procedures. Mice were monitored daily for the development of neurological signs and body weight loss. Animals reaching endpoint criteria were transcardially perfused using 2% paraformaldehyde and the brains removed for histology. Tissue preparation and cryosectioning was performed as described by Jimenez-Pascual *et al.*^31^ Briefly, 30 μm thick sections were cut on a cryomicrotome and stored in cryoprotectant until use. For CARS microscopy, tissue sections were mounted onto glass slides and air dried before being reconstituted with double-distilled water (ddH_2_O). Prior to imaging, sections were covered with a #1.5, 24 × 24 mm coverslip (Menzel Gläser, Agar Scientific AGL46S24-15) and sealed with nail varnish. Endogenous eGFP expression in GBM cells was used to discriminate between tumour and host cells.

### 2.3. Optical micro-spectroscopy

Imaging of both the living organoids and fixed glioblastoma tissues was performed on the same inverted microscope (Nikon, Eclipse Ti-U). Alongside conventional wide-field *epi*-fluorescence and DIC imaging, the microscope has been modified to enable two-photon fluorescence (TPF) and hyperspectral CARS imaging modalities, described in our previous work.^8^ A home-built environmental chamber surrounds the microscope allowing a constant temperature of 37 °C with a 5% CO_2_/air-blend environment to be maintained for live cell imaging. Both sets of measurements used a 40 × 1.15 NA water-immersion objective (Nikon, MRD77410 CFI Plan Apochromat λS series) with a 1.0 × tube lens. A 1.34 NA oil-immersion condenser lens (Nikon, MEL41410) was used with the fixed glioblastomas, while a 0.72 NA dry condenser (Nikon, MEL56100) was used with the organoids to accommodate the low profile dish and lid of the CO_2_ chamber. *xy* sample motion and *z* objective motion are automated by stepper motors (Prior, ProScan III controller (V31XYZ), *xy* stage (H117NN2N) and *z* drive (PS3H122)).

#### 2.3.1. Wide-field DIC and *epi*-fluorescence microscopy

A CCD camera (Hamamatsu, Orca 285) with 1344 × 1024 pixels 6.45 μm in size is used for both DIC and fluorescence acquisition. Considering the 40× magnification, the pixel size corresponds to 0.163 μm at the sample plane. The camera has 9 e read noise and 18 ke full-well capacity. The images are digitized by a 12-bit A/D converter and saved to 16-bit greyscale TIFF format.

DIC illumination was provided by a halogen tungsten lamp (Nikon, V2-A LL 100 W) followed by a blue-green filter (Schott, BG40) to block near-infrared light, for which the DIC polarisers do not have sufficient extinction. A red bandpass filter (ThorLabs, FB650-40 (650 ± 20) nm) is used to define the wavelength range; the longer (red) wavelengths penetrate further into the organoids and do not excite fluorescence, avoiding photobleaching. A de Sénarmont compensator (Nikon, MEN51941 T-P2 DIC Polarizer HT) is used for DIC offset phase adjustment, consisting of a polarizer and a quarter-wave plate in the illumination beam path. The angle *θ* of the polarizer with respect to the fast axis of the quarter-wave plate is adjustable. The compensator is followed by a Nomarski prism in the condenser unit (Nikon, MEH52500 HNA N2 Oil, with the 1.34 NA oil-immersion condenser, or Nikon, MEH52400 CLWD N2, with the 0.72 NA dry condenser). A second Nomarski prism is placed after the objective (MBH76245 D-C DIC Slider 40X-II, Nikon), followed by a linear polariser (Nikon, MEN51980 Ti-A-E DIC Analyzer Block) in the filter turret. DIC phase angle setting for the organoids was *θ* = 45°, and for the brain tissues was *θ* = 0° (no background transmission) and *θ* = 40°. An exposure time of 0.1 s was used for all DIC experiments. The lamp illumination intensity was set to result in a maximum image pixel value around 15 ke, corresponding to about 2 mW cm^−2^ at the sample for the 40° and 45° rotation angles and about 70 mW cm^−2^ for the 0° rotation angle.

For the brain tissue samples, wide-field *epi*-fluorescence excitation of eGFP was provided by a metal–halide lamp (Prior Scientific, Lumen L200/D) set at 10% of the maximum power. A suitable exciter/emitter/dichroic filter cube (Semrock, GFP-A-Basic) was used, resulting in an illumination intensity of about 3 W cm^−2^ at the sample. Fluorescence images were acquired with an exposure time of 0.03 s.

#### 2.3.2. TPF and hyperspectral CARS microscopy

**Fig. 1:**
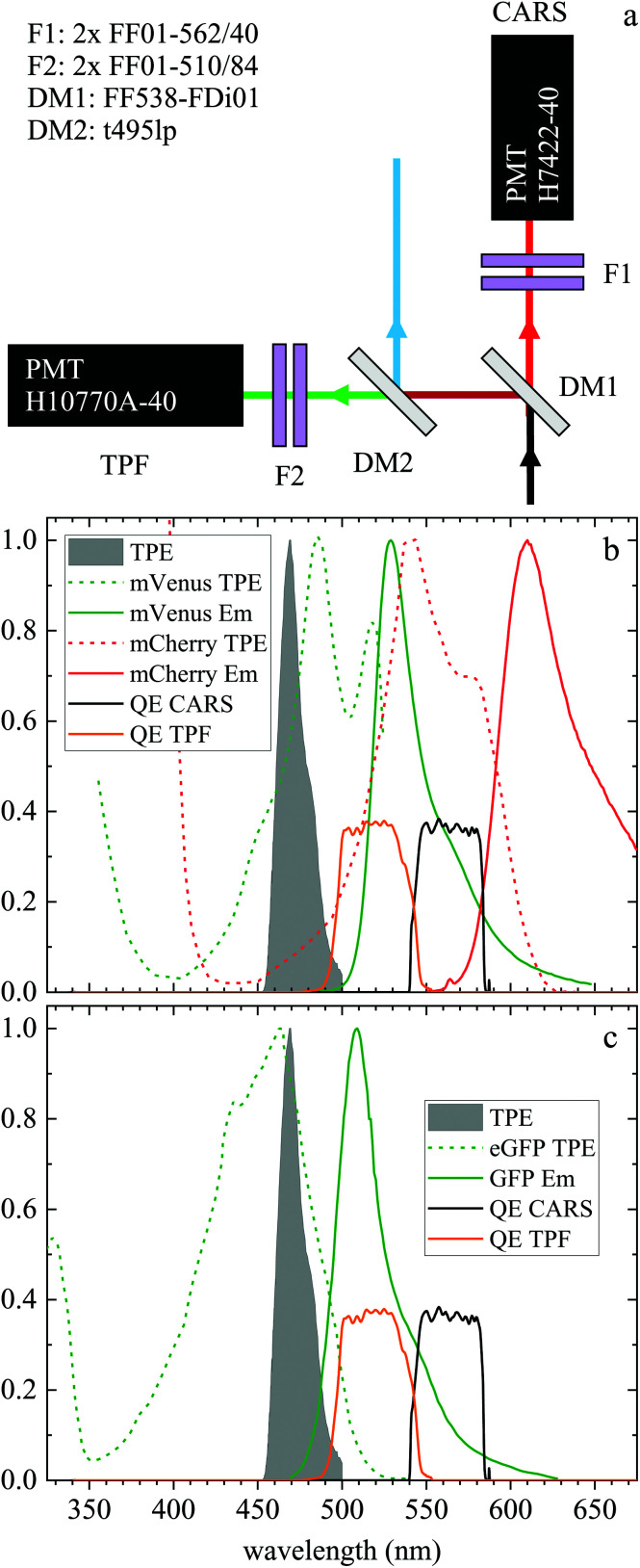
(a) Forward detection layout. A dichroic beamsplitter (DM1) transmits the CARS emission at wavelengths >540 nm and reflects the TPF emission at shorter wavelengths. In the CARS detection path, a pair of bandpass filters (F1) rejects unwanted signals from reaching the CARS PMT. The signal reflected by DM1 travels through a second dichroic mirror (DM2) which reflects wavelengths below 495 nm. A pair of bandpass filters (F2) rejects unwanted signals from reaching the TPF PMT. (b) Normalized two-photon excitation (dashed lines) and emission (solid lines) spectra for the mVenus^32^ (green) and mCherry^33^ (red) fluorescent proteins in the Fucci2aR system. (c) Normalized two-photon excitation^33^ (dashed line) and emission (solid line) spectrum of eGFP used in the glioblastoma tumour cells. Also plotted are the normalized TPF excitation pulse spectrum (grey infill curve, at half its wavelength) and the quantum efficiency of the TPF (orange) and CARS (black) PMT detection from the entrance into DM1.

Two-photon fluorescence and hyperspectral CARS microscopy images were acquired using the experimental setup described in our previous work^8^ with some minor upgrades, as indicated below. Briefly, we use a single broadband 5 fs pulsed lasersource, with a pulse spectral width of 310 nm at 10% of the maximum intensity (660 nm to 970 nm) at a repetition rate of 80 MHz (Venteon, Pulse:One PE). A long-pass filter consisting of two 2.5 mm thick Hoya R66 filters at Brewster angle rejects the short wavelength laser beam tail, having a steeper cut-off than the Schott RG645 used previously. Dichroic beamsplitters separate the laser beam into the pump and Stokes beams for CARS (CVI Melles Griot, LWP-450RP670-TP830-PW-1025-C), and a third beam (Eksma Optics, custom short-pass) for two-photon excitation (TPE) of fluorescence. The pulses are peaked at 685 nm (pump), 806 nm (Stokes) and 940 nm (TPE), with a bandwidth at 10% intensity of 65 nm, 200 nm and 70 nm, respectively. A prism-based pulse compressor is used to obtain TPE pulses with a Fourier-limited pulse duration of approximately 30 fs at the sample. Spectral focussing with glass elements^34^ is implemented to achieve narrow-band CARS spectral resolution and wavenumber tuning from the broadband pulses. Vibrational frequencies between 1200 and 3800 cm^−1^ can be addressed by varying the instantaneous frequency difference (IFD) between pump and Stokes pulses, which is controlled by adjusting the relative delay time between the pulses with an optical delay line (PI, M-404.42S). Additional glass elements in the Stokes beam path tune the Stokes linear chirp to match that of the pump pulse, enabling a high spectral resolution of 10 cm^−1^ to be achieved. The pump, Stokes and TPE beams are recombined using the same dichroic beam-splitters used to split them prior to entering the scan mirrors (Cambridge Technology, 6210HSM40). A scan lens (from a Nikon, A1RMP multiphoton microscope) is used to focus the collimated beam from the scan mirrors into the intermediate image plane at the microscope port, and image the scan mirrors onto the back focal plane of the microscope objective. Signals are collected in the forward direction by the condenser lens and detected as shown in Fig. 1(a). A dichroic beamsplitter DM1 (Semrock FF538-FDi01) transmits CARS at wavelengths >540 nm and reflects signals at shorter wavelength. In the CARS detection path, a pair of bandpass filters F1 (Semrock FF01-562/40 for the 2500–3800 cm^−1^ range) rejects the excitation laser (by more than 13 orders of magnitude) and the transmitted light is detected by the CARS photomultiplier (PMT; Hamamatsu, H7422-40). The signal reflected by DM1 travels through a second dichroic beamsplitter DM2 (Chroma t495lp) which reflects wavelengths below 495 nm, such as second harmonic generation of the TPE beam^8^ (not used here), and transmits TPF. A pair of bandpass filters F2 (Semrock FF01-510/84) rejects the excitation laser and transmits the TPF to the PMT (Hamamatsu, H10770A-40). DM1 and the TPF PMT have been upgraded from our original setup description.^8^ The resulting quantum efficiency spectra of the CARS and TPF PMT detection channels are shown in Fig. 1 together with the relevant two-photon absorption and emission spectra of the fluorophores used.

##### 2.3.2.1. Organoid acquisition settings

Organoids were first located using DIC, as described above. TPF and hyperspectral CARS images were acquired sequentially to avoid bleaching of TPF by the CARS excitation, as well as bleed through of TPF into the CARS detection, see results section for discussion. TPF images and hyperspectral CARS z-stacks were acquired at 1 μm and 5 μm intervals respectively, over a *z*-range of 30 μm. The pixel size in the *x*, *y* plane was (86 nm)^2^. A pixel dwell time of 100 μs and 1 μs and PMT gains of 10^6^ and 10^4.6^ were used for TPF and CARS, respectively. Typical excitation powers at the sample were 41 mW (pump), 31 mW (Stokes) and 11 mW (TPF). Hyperspectral (*x*, *y* and wavenumber) CARS scans were acquired over the range 2600–3800 cm^−1^, with 5 cm^−1^ step size. Following each scan, an image was acquired with the pump and Stokes pulses out of time overlap (all other settings identical) to measure non-CARS background.

##### 2.3.2.2. Brain tissue acquisition settings

Wide-field fluorescence and DIC were first used to locate GSCs and NGCs within the brain section. TPF and hyperspectral CARS images were acquired sequentially to avoid bleed through of the strong fluorescence signal into the CARS detection, see results section for discussion. PMT gains of 10^4.8^ and 10^5^ were used for TPF and CARS, respectively with a pixel dwell time of 10 μs for both. The pixel size in the *x*, *y* plane was (80 nm)^2^. Typical excitation powers at the sample were 31 mW (pump), 23 mW (Stokes) and 8 mW (TPF). Hyperspectral CARS scans were acquired over the range 2600–3700 cm^−1^, with 5 cm^−1^ step size. Following each hyperspectral CARS scan, an image was acquired with the pump and Stokes pulses out of time overlap (all other settings identical) to determine background offset levels.

### 2.4. CARS hyperspectral analysis

Hyperspectral CARS datasets can be difficult to visualise and interpret, and for this purpose we have developed the Hyperspectral Image Analysis (HIA) software.^9^ HIA enables the retrieval of the imaginary part of the CARS susceptibility 
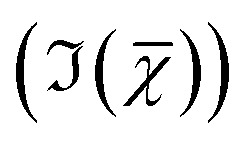
 and the factorisation of the spectra into a specified number of chemical components with corresponding concentration maps. Briefly, the HIA pipeline operates as follows.^9,35,36^ Firstly, a background image acquired with the pump and Stokes beams out of time overlap is subtracted from each image of the hyperspectral CARS scan. As well as subtracting backgrounds *e.g.* from the electronics, this step also removes potential fluorescence contributions generated from the individual pulses. Singular-value decomposition (SVD) is then employed to denoise the data. In order to correct for the varying temporal overlap of the pump and Stokes beams, data are divided by the spectrum of a non-resonant material (glass), acquired with the same laser power and acquisition settings. If there is a region filled with water present in the image, this may be used as reference material, combined with a previously calibrated glass/water ratio. If there is no such region present (as with the brain tissue samples), a glass spectrum is measured in the glass coverslip immediately after the acquisition of the CARS data on the sample. To enable quantitative analysis, the imaginary part of the CARS susceptibility 
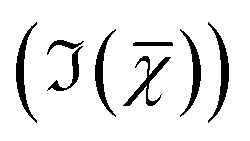
 is retrieved using a phase-corrected Kramers–Kronig (PCKK) procedure, which recovers a Raman-like spectrum. Finally, the retrieved spatially-resolved susceptibility spectra are factorised into spectra and spatially-resolved concentration maps of chemical components (a method which we called FSC^3^), based on non-negative matrix factorisation. We use an iterative algorithm^37^ where concentration maps and spectra are sequentially varied to minimise the factorisation error. Importantly, the factorisation is unsupervised, *i.e.* it does not require prior knowledge of the sample's chemical composition. The number of components used in the factorisation can be varied, with the optimal number being the minimum required to provide unique spatial and spectral features. Typically, in the range of 6 to 7 components are needed, with 5 to 6 components being physically meaningful, and 1 or 2 components accounting for spurious noise and imaging artefacts. To reduce the importance of water, which is dominating the 
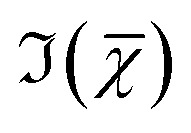
 spectra above 3100 cm^−1^, the spectral range analysed by FSC^3^ is typically limited^11^ to a maximum of 3150 cm^−1^, which retains the relevant vibrational resonances of the organic material. FSC^3^ may be applied to hyperspectral data sets individually, or multiple sets can be analysed together. It is also possible to take components from a selected factorisation analysis, and use these as a base to factorise other data sets. This is referred to as projection, and uses a single step of the factorisation algorithm which calculates the concentration maps, while keeping the spectra fixed to the given base.

## 3. Results and discussion

### 3.1. Fucci2aR liver bile duct organoids

**Fig. 2:**
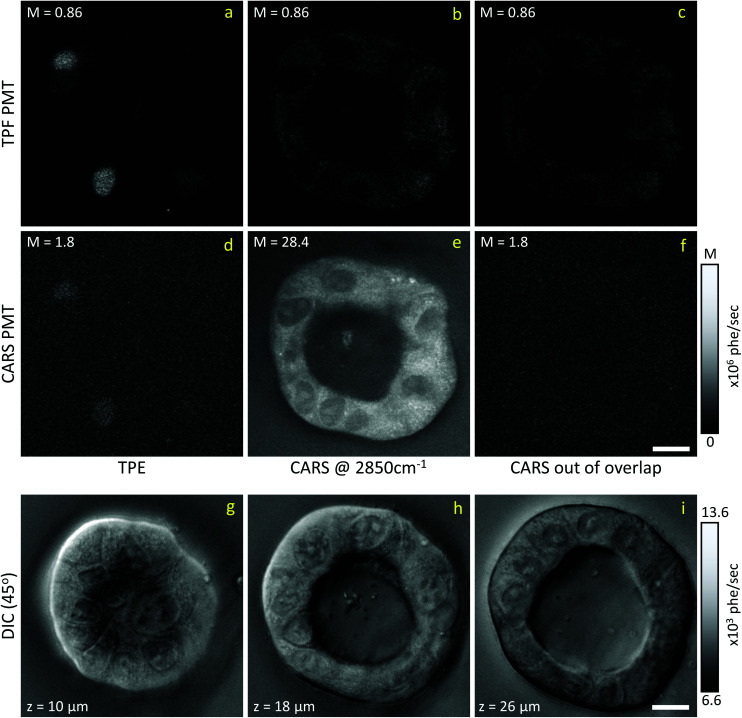
Examples of CARS and TPE excitation and detection data on living Fucci2aR liver bile duct organoids. (a–c) Signal detected by the TPF PMT; (d–f) signal detected by the CARS PMT; (a and d) TPE excitation; (b and e) CARS excitation at 2850 cm^−1^ IFD; (c and f) CARS excitation with pump and Stokes pulses out of time overlap. Image grey scale from 0 to *M* × 10^6^ photoelectrons per second as indicated. (g–i) DIC images (+45° polariser rotation angle) taken at three different *z* positions (see inset in image for *z* value) through the organoid, *z* = 0 μm is defined as the bottom of the organoid. The *z* position of the CARS/TPF data shown above is approximately 15 μm, however DIC images are taken 30 min earlier, prohibiting an exact correlation due to organoid development. Images grey scale as given in photoelectrons per second per pixel. Scale bars 10 μm.

Liver organoids investigated here incorporated the fluorescence ubiquitination-based cell cycle indicator (Fucci2aR) system (see section 2.1.1). This is a fluorescent protein-based construct that employs a red (mCherry) and a green (mVenus) fluorescent protein fused to two cell cycle dependent proteins, Cdt1 and geminin, respectively. In eukaryotic cells, Cdt1 level peaks during G1 phase and plummets upon S entry. Conversely, the geminin level is high during S, G2 and M phases, and is degraded in the subsequent G1 phase. Therefore, when cells are in the G1 phase, the high level of mCherry gives rise to red-fluorescent cell nuclei, whilst in the S/G2/M phase the high level of mVenus results in green-fluorescent nuclei. We used this construct as a fluorescence marker, enabling direct correlation of TPF and hyperspectral CARS imaging on the same living organoid, to investigate whether hyperspectral CARS is able to distinguish subpopulation of cells at different phases in the cell cycle. The experiment was set-up to avoid bleed-through between fluorescence emission and CARS detection, as detailed in the following. The two-photon excitation and emission spectra for mCherry^33^ and mVenus^32^ are displayed in Fig. 1(b) overlaid with the spectrum of the TPE beam, and with the TPF and CARS PMT detection quantum efficiency (QE) spectra for the optical setup. mCherry is excited only weakly by the TPE beam, and its emission lies above the detection ranges of both the TPF and CARS PMTs. Therefore, no fluorescence signal is observed from cells in the G1 phase under these excitation/detection conditions. Conversely, the mVenus TPE spectrum overlaps well with the TPE pulse spectrum, and the peak of its emission falls within the detection range of the TPF PMT. There is also an overlap of the mVenus emission spectrum with the CARS PMT detection range. Under TPE beam excitation, we therefore observe a strong signal detected by the TPF PMT, and a weaker signal from the CARS PMT, from those cells that are in the S/G2/M phase of their cell cycle. This is demonstrated by the images in Fig. 2(a and d) which show the signal in an *xy* plane sectioning the organoid, detected by the TPF and CARS PMTs when the sample is excited only by the TPE beam. For comparison, Fig. 2(b and e) illustrates the detected signal when the sample is excited only by the CARS (pump and Stokes) beams at 2850 cm^−1^ IFD. Here, we observe a strong signal in the CARS PMT from lipids and proteins in the cell cytoplasm, with the cell nucleus appearing dark. The organoid forms a hollow structure, as can also be seen in the DIC images (Fig. 2(g–i)) taken at different *z*-planes through the organoid. A signal from the cell cytoplasm is observed in the TPF PMT under CARS excitation. This is originating from two-photon excited auto-fluorescence, as can be seen in Fig. 2(c and f) which illustrates the observed signal when the sample is excited by the pump and Stokes beams out of time overlap (*i.e.* there is no CARS signal generated). Such autofluorescence is predominantly generated by the pump beam, considering the pump pulse TPE peak at around 340 nm.^38^Fig. 2(c and f) also demonstrates that there is no fluorescence detected by the CARS PMT under pump and Stokes excitation when the pulses are not in temporal overlap. To maintain such de-coupling, TPF and CARS images were acquired sequentially, rather than simultaneously, throughout the experiments.

**Fig. 3:**
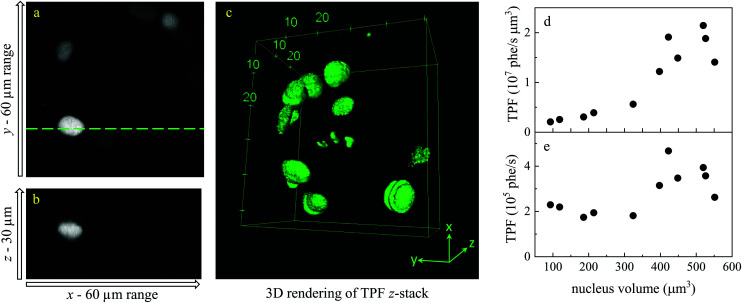
TPF *z*-stack of a Fucci2aR liver bile duct organoid, showing mVenus as a marker of the S/G2/M phase in the cell cycle. (a) Single *xy* plane. (b) *zx* plane at the line indicated in the *xy* plane. Images are shown on an intensity grey scale from 0 to 1.2 × 10^6^ photoelectrons per second. (c) 3D rendering of the TPF *z*-stack. (d and e) TPF *versus* nucleus volume for each fluorescent cell across the 3D-stack, shown as detected photon rate integrated over each nucleus volume (d) and as average photon rate over the nucleus volume (e).

TPF images were acquired in 3D, as *z*-stacks with 1 μm intervals, while hyperspectral CARS *z*-stacks were acquired at 5 μm steps through the organoid (see section 2.3.2.1). This choice was made in order to compromise between 3D imaging and the time required for hyperspectral CARS acquisition, while minimising motion artefacts and photodamage, since organoids were alive. The TPF *z*-stack is used to positively identify the cells in the S/G2/M phase of their cell cycle and generate a 3D reconstruction of their position for correlation with the hyperspectral CARS data. A *xy* slice with *z*-projection, along with a 3D rendering of the TPF *z*-stack is presented in Fig. 3. An overview of all *xy* planes is shown in Fig. S1.† The TPF volumetric dataset was then quantitatively analysed, as follows. Cell nuclei volumes were calculated from the 3D reconstruction using the *3D Objects Counter* plugin^39^ for the image analysis software ImageJ to segment the nuclei (see section S1 ii and Fig. S2†). The fluorescence intensity integrated over the nucleus volume is given in Fig. 3(d)*versus* nucleus volume for each cell which showed localization of TPF in the nucleus. A clear trend of increasing fluorescence intensity with increasing nucleus volume is observed, consistent with the progressive increase of the size of a cell and its nucleus during the eukaryotic cell cycle,^40–42^ and that mVenus-geminin is highly expressed in the later phases of the cycle, once the cell has started its DNA replication. Notably, we observe a step in the intensity increase for intermediate cell volumes between 300 μm^3^ and 400 μm^3^, which is clearly visible in the average intensity over the nucleus volume, as shown in Fig. 3(e). The correlation between nucleus size and mVenus fluorescence intensity suggests that low-intensity small-nucleus cells are at the G1/S phase, intermediate sized nuclei with high intensity are in the S/G2 phase, and large nuclei are in the G2/M phase.

**Fig. 4:**
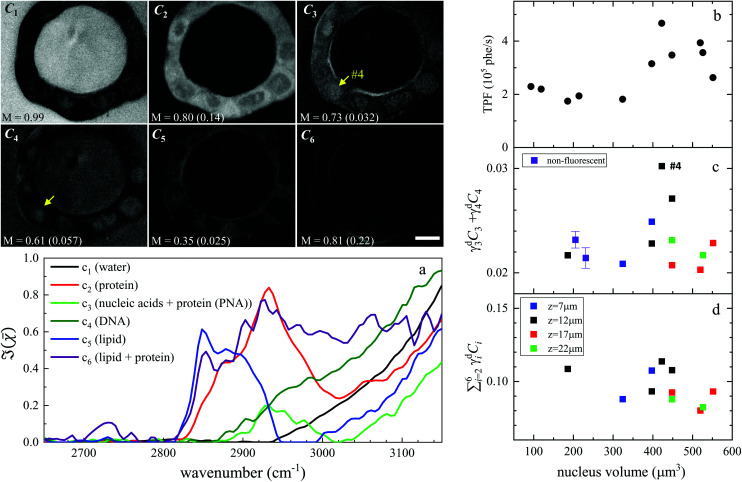
Overview of the FSC^3^ analysis on Fucci2aR liver bile duct organoids. Images *C*_1_ to *C*_6_ are the concentration maps of the corresponding components, on a grey scale from 0 to *M* as indicated in vol/vol units. The corresponding dry vol/vol concentrations are given in parenthesis. Images are shown for the plane corresponding to the TPF *xy* section in Fig. 3, and the strongly fluorescent cell visible in the TPF section is indicated as #4, for reference. (a) Susceptibility spectra of the FSC^3^ components. (b–d) Dry concentrations of components averaged over the nuclei areas, for the cells that exhibited mVenus TPF in the nucleus, as a function of the nucleus volume (each data point represents an individual nucleus). Different *z*-planes were evaluated (indicated by colours), and data are shown for the planes showing the largest nucleus area (see text). Purple symbols show the mean (bar: standard error of the mean) of the dry components for two sets of cells having no detectable mVenus fluorescence in the nucleus (see text). The TPF intensity per nucleus volume is plotted again (same data as in Fig. 3), for direct comparison. Scale bar 10 μm.

Hyperspectral CARS was quantitatively analysed using the HIA/FSC^3^ pipeline as described in section 2.4. The resultant susceptibility spectra and concentration maps measured at the same *xy* plane as shown in Fig. 3 are presented in Fig. 4. Seven spectral components were used. Based on the retrieved susceptibility spectra and the spatial profile of the concentration maps, the first six components were attributed to c_1_: water, c_2_: protein, c_3_: nucleic acids + protein (PNA), c_4_: DNA, c_5_: lipid, c_6_: lipid + protein. This is consistent with published reports^9,11,43^ showing that: (i) water has a Raman spectrum peaked at around 3300 cm^−1^, hence appears as a tail in the 2800–3200 cm^−1^ range; (ii) the ratio between the CH_2_ symmetric stretch vibration (2850 cm^−1^) and the CH_3_ resonance (2930 cm^−1^) discriminates between lipids (rich in CH_2_ bonds) and proteins (peaked at 2930 cm^−1^); (iii) nucleic acids exhibit Raman spectra peaked at 2950 cm^−1^ and DNA is confined to the cell nucleus. We note that all components contain a significant amount of water additional to the attributed organic material. c_4_ is dominated by a water contribution. The water-subtracted spectrum of this component (see Fig. S9†) shows a spectral distribution between 2900 and 3100 cm^−1^ consistent with the main features of DNA (see ref. 43Fig. 1B). The spectral shape and concentration map of c_7_ indicates that this is not a physically meaningful chemical component, and is attributed to imaging or sample motion artefacts (see Fig. S3, and S6† for an overview of concentration and spectral errors). Inspection of the concentration maps reveals that there is no obvious chemical component that can be directly correlated with the mVenus marker. Indeed, while during the cell cycle it is known that the amount of proteins and nucleic acids is growing in preparation for mitosis, at the same time, the cell increases its volume at a steady rate, hence it is unclear whether the concentration of these components should be significantly altered during the cycle. On the other hand, it has been shown, using yeast as model systems of the eukaryotic cell cycle,^41,42^ that despite the sharp increase in DNA copy number during DNA replication in the S-phase, the size of the cell nucleus does not abruptly increase in parallel, suggesting that the DNA concentration could be significantly higher at the end of the S phase.

To investigate this point, we analysed all available hyperspectral CARS datasets for the different *z*-planes, using the seven components shown in Fig. 4 as common basis (projection analysis, see Materials and methods). On these data sets, we identified those cells that exhibited TPF of mVenus in the nucleus, and calculated the average concentration of each component over the nucleus area. Since hyperspectral CARS *z*-stacks were performed with coarse 5 μm steps, well above Nyquist sampling, we could not examine the retrieved concentration components volumetrically in 3D. For each cell, we therefore selected the *z*-planes where the nucleus area appeared largest, as to avoid *z*-planes too close to the nucleus top/bottom edges (see also Fig. S10†). This is to ensure that the measured concentrations are only sampling the nucleus region, considering the 1.5 μm wide axial point-spread function of 
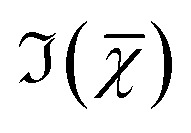
 for the data shown. Concentrations of c_2_ to c_6_ were scaled to represent dry values, by removing the water contribution, as described in our recent work.^11^ Briefly, for each component, we calculated the dry volume fraction *γ*^d^_*i*_, to obtain the corresponding dry vol/vol concentration *γ*^d^_*i*_*C*_*i*_. Details on the determination of *γ*^d^_*i*_ are given in section S1 iv.† Fig. S11† shows *γ*^d^_*i*_*C*_*i*_ averaged over the nucleus area, for each cell that exhibited TPF of mVenus in the nucleus, taken at the selected *z*-plane (as explained above), as a function of the nucleus volume. Since c_3_ and c_4_ both are localized in the nucleus and have a spectrum comprising DNA, we considered the sum of these two components, which is shown in Fig. 4(c). Notably, we see that this sum *γ*^d^_3_*C*_3_ + *γ*^d^_4_*C*_4_ sharply increases with increasing nucleus volume around 350 μm^3^, following a similar trend as the TPF intensity, which we attributed as signature of the S/G2 phase. The subsequent decrease of this concentration for large nuclei volumes in the G2/M phase, when the DNA replication has ended, is in line with the expansion of the nucleus size in preparation for mitosis while no longer increasing its DNA content. The spread of concentration values for different *z*-planes of one cell around 450 μm^3^ nucleus volume could also be indicative of an asymmetric distribution of DNA in preparation for mitosis. The total dry concentration 
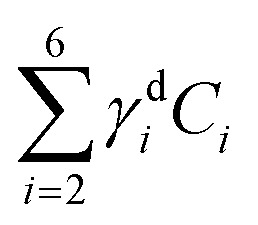
 is shown in Fig. 4(d), and appears approximately constant *versus* nucleus volume. The overview of each component given in Fig. S11† shows that individual components do not exhibit a well defined trend. Indeed, only *γ*^d^_3_*C*_3_ + *γ*^d^_4_*C*_4_ appears to correlate with the TPF intensity dependence, suggesting that this combination could be used as a marker of the S/G2 phase in the cell cycle. For consistency, we also examined the cells imaged by hyperspectral CARS that did not exhibit a significant mVenus TPF in the nucleus. Fig. 4(c) shows the mean of *γ*^d^_3_*C*_3_ + *γ*^d^_4_*C*_4_ over these cells (purple data points). We considered two cases: all the non-fluorescent nuclei areas, and a sub-set whereby we excluded nuclei with very small areas (< 22 μm^2^), which could be too close to the top/bottom nucleus edges, as well as nuclei with large areas (> 53 μm^2^), to exclude cells beyond the G1 phase. The two purple data points represent the mean for these two sets. An overview of *γ*^d^_3_*C*_3_ + *γ*^d^_4_*C*_4_ for each cells in the two sets, and the resulting mean, standard deviation, and standard error of the mean is shown in Fig. S12.† For the non-fluorescent cells we used the nucleus area *A* to calculate the volume as *V* = *A*^3/2^, to position these data points in Fig. 4. Considering all non-fluorescent cells, we obtained a mean of *γ*^d^_3_*C*_3_ + *γ*^d^_4_*C*_4_ which is slightly higher than when selecting cells as described above (see also Fig. S12†), but in both cases the values are below those observed for the fluorescent cells attributed to the S/G2 phase, and in line with the lower values seen in the other phases, consistent with our interpretation. It should be noted that the limited number of fluorescent cells available in this study implies that the analysis has a modest statistical significance, hence the results are to be taken as indicative. The emphasis here is on the quantitative data analysis pipeline presented, offering a valuable methodology for investigating quantitative chemically signatures of the cell cycle with hyperspectral CARS microscopy.

### 3.2. Brain tissue with GBM

**Fig. 5:**
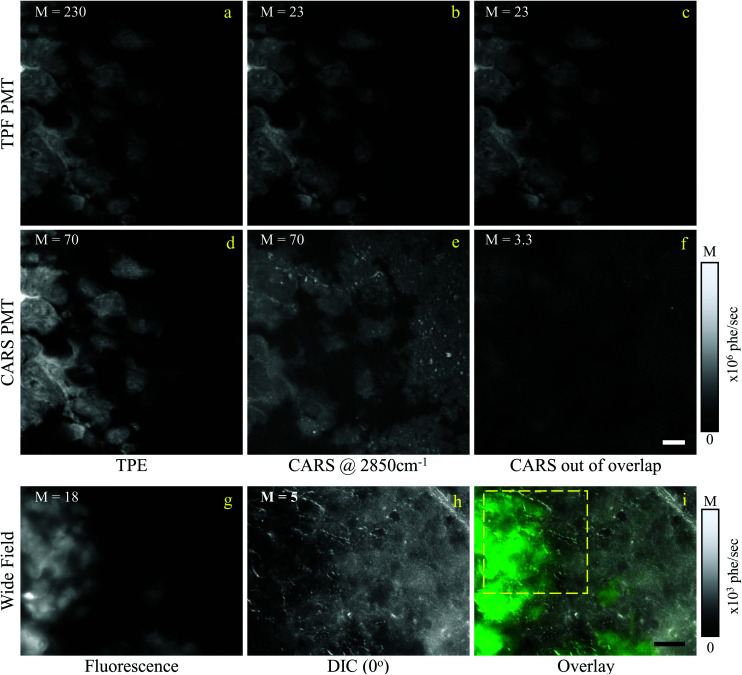
Overview of the CARS and TPF excitation and detected configuration for a mouse brain tissue, following implantation of eGFP-labelled GSCs in mice (see text). (a–c) Signal detected by the TPF PMT; (d–f) signal detected the CARS PMT; (a and d) TPF excitation; (b and e) CARS excitation at 2850 cm^−1^ IFD; (c and f) CARS excitation with the pump and Stokes pulses out of time overlap. Images are shown on a grey scale from 0 to *M* × 10^6^ photoelectrons per second as indicated. Scale bars: 10 μm. (g) Wide field *epi*-fluorescence of eGFP; (h) DIC image (0° polariser rotation angle); (i) overlay of wide field fluorescence (green) and DIC (grey). Scale bar 30 μm. The dashed yellow frame indicates the ROI imaged with CARS and TPF.

In this section, we report our work on brain tissues obtained from mice xenografted with either eGFP-labelled GBM stem cells (GSC) or eGFP-labelled non-stem GBM cells (NGC). The aim was to investigate if hyperspectral CARS microscopy can separate normal brain tissue from cancerous cells, and if tumours derived from GSC *versus* NGC can be distinguished. eGFP labelling enables positive correlation with CARS imaging. As discussed in section 3.1, signal bleed through between CARS and TPF can occur and needs to be corrected for. The two-photon excitation and emission spectra for eGFP are displayed in Fig. 1(c) together with the spectra of the TPE beam and the TPF and CARS PMT QE. The TPE beam spectrum overlaps well with the eGFP excitation spectrum, and the eGFP emission overlaps well with the TPF PMT QE. There is also a significant overlap of the eGFP emission with the CARS PMT QE. We can therefore expect to see eGFP emission in both the TPF and CARS PMTs. This is confirmed experimentally, as reported in Fig. 5(a and d), showing the TPF and CARS PMT signals when the sample is excited only by the TPE beam. Fig. 5(b and e) illustrates the detected signal when the sample is excited only by the CARS (pump and Stokes) beam, at 2850 cm^−1^ IFD. We observe a strong signal in the CARS PMT from lipids and proteins in the cell cytoplasm. In the TPF PMT, in addition to the two-photon excited auto-fluorescence, predominantly generated by the pump pulse (similar to what described in section 3.1), we also see a substantial contribution from the eGFP emission. This is a result of the significant spectral overlap of the TPE by the pump beam around 340 nm and the two-photon excitation of eGFP (see Fig. 1). Fig. 5(c and f) shows the observed signal when the sample is excited by the pump and Stokes beams out of time overlap. We can see a weak fluorescence detected in the CARS PMT, due to two-photon excitation from the individual pump and Stokes pulses. Although about 20 times weaker than the CARS signal, this contribution is taken into account in the HIA/FSC^3^ pipeline analysis as described in section 2.4. In the experiments, TPF excited by the TPE beam and CARS excited by the CARS beams were acquired sequentially.

**Fig. 6:**
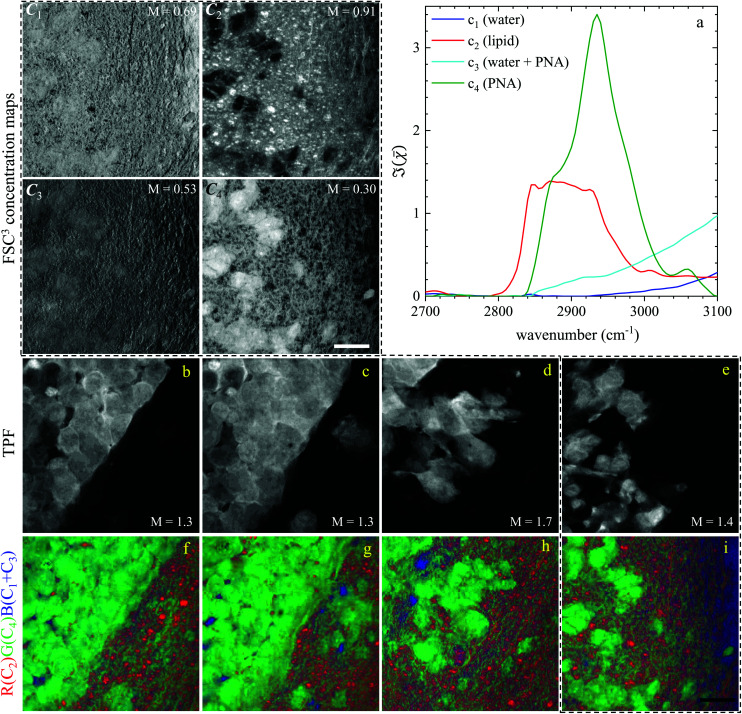
Overview of the hyperspectral CARS FSC^3^ analysis for GSC-derived tumour cells, showing concentration maps (in vol/vol units) of the spectral components. (a) Component spectra. (*C*_1_–*C*_4_) concentration maps of the components from one ROI, grey scale from 0 to *M* as indicated in vol/vol units. (b–e) TPF images of four ROIs identifying GSC-derived cells; grey scale range 0 to *M* × 10^8^ photoelectrons per second. (f–i) False colour maps generated from the overlay of concentrations of components c_2_ (red), c_4_ (green), c_1_ + c_3_ (blue). All images are on the same RGB scale, from 0 to a colour maximum scale *M* (vol/vol) with *M*_red_ = 0.78, *M*_green_ = 0.32, *M*_blue_ = 1.48. Scale bar: 20 μm.

Regions of interest (ROIs) containing both cancer cells and normal tissue were initially found using wide-field *epi*-fluorescence and DIC imaging, see Fig. 5(g–i) for tissues from mice implanted with GSC cells. A *z*-position approximately in the centre of the 30 μm thick section was located using TPF imaging. TPF and hyperspectral CARS scans were acquired on multiple ROIs (6 for GCS-derived tumours and 2 for NGC-derived tumours) as described in section 2.3.2.2. Hyperspectral CARS scans were then processed through our HIA/FSC^3^ pipeline, as described in section 2.4. For these samples, using five components in the FSC^3^ was found to be optimal. All 6 ROIs in tissues from mice implanted with GSC cells were analysed together, hence spectra are a common basis in the factorisation. Exemplar concentration maps for one of the ROIs are shown in Fig. 6(*C*_1_–*C*_4_) in grey scale (all ROIs are given in Fig. S13 and S15–S20†).

Based on the spectral shape and the spatially-resolved concentration maps, the components were assigned to c_1_: water, c_2_: lipid, c_3_: water + PNA, c_4_: PNA. Component c_5_ exhibited a spectrum that is not physically meaningful, and thus is attributed to artefacts (see Fig. S14†). Fig. 6 shows the susceptibility spectra and concentration maps of components c_1_ to c_4_ for the GSC-derived tumour cells. RGB overlays of the concentration maps of components c_2_ (red), c_4_ (green) and c_1_ + c_3_ (blue) are presented in Fig. 6(f–i) for four ROIs. Comparing them with the corresponding TPF images shown in the figure on the row above, there is a clear separation between the GSC-derived cancer and normal brain tissue. Tumour cells in the colour concentration maps appear green-blue, hence richer in water and proteins compared to normal tissue. Notably, this finding is consistent with the fact that tumour cells proliferate faster than normal cells, hence they need to make more proteins, a point well recognised in the cell biology literature (for a review see ref. 44). It is also known that malignant tissue contains more water than normal tissue. Some of this water is between cells (interstitial), but there are reports^45^ showing that tumour cells contain more water than normal tissue. This could be explained since, in most instances, tumour cells are larger than normal cells, albeit this point is not yet fully understood.

**Fig. 7:**
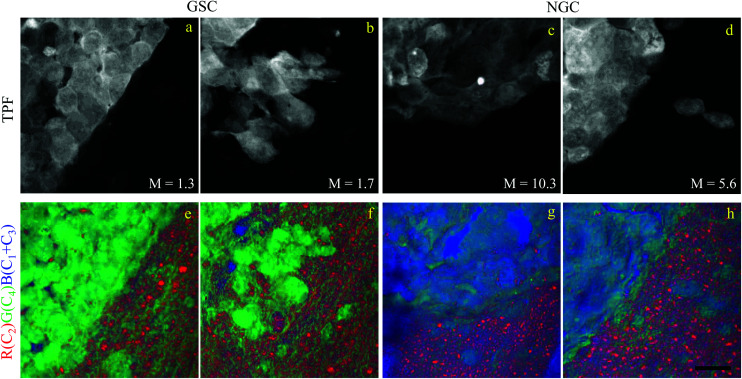
Comparison of the hyperspectral CARS FSC^3^ analysis on brain sections with GSC-derived and NGC-derived tumours. Samples with NGC-derived tumours were analysed using as spectral basis the 5 components of the FSC^3^ analysis from samples with GSC-derived tumours (see Fig. 6). (a–d) TPF images identifying GSC-derived and NGC-derived cancer cells. Intensity grey scale from 0 to *M* × 10^8^ photoelectrons per second. (e–h) False colour maps generated from the overlay of the concentrations of components c_2_ (red), c_4_ (green), c_1_ + c_3_ (blue). RGB channel range 0 to *M* (vol/vol) adjusted as in Fig. 6. Scale bar 20 μm.

**Fig. 8:**
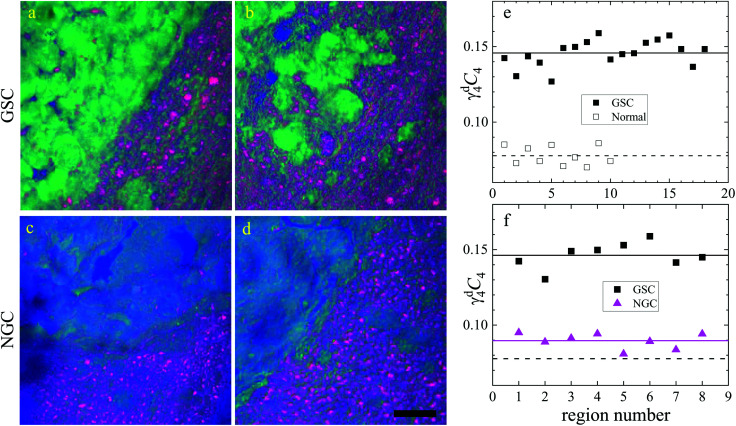
Dry component analysis on brain sections with GSC-derived and NGC-derived tumours. False colour dry concentration maps of the FSC^3^ components lipid *γ*^d^_2_*C*_2_ (red) and PNA *γ*^d^_4_*C*_4_ (green), and the total water concentration 
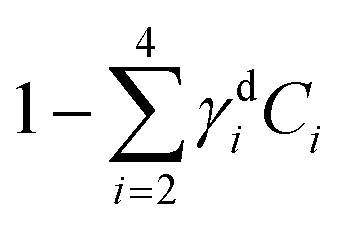
 (blue). (a and b) GSC and (c and d) NGC samples. Images are on the same RGB scale, from 0 to a colour maximum scale *M* (vol/vol) with *M*_red_ = 0.18, *M*_green_ = 0.22, *M*_blue_ = 1.48. Scale bar: 20 μm. (e and f) Dry PNA concentration of component *γ*^d^_4_*C*_4_ spatially averaged over selected areas identified from TPF and false colour maps (see Fig. S24 and S25† for region maps). (e) GSC-derived tumour regions (solid black squares) compared with regions of normal tissue (hollow squares). The lines show averages for GSC-derived tumour regions (solid); and normal tissue regions (dashed). (f) GSC-derived (black squares) and NGC-derived (magenta triangles) tumour regions for the two GSC and two NGC ROIs shown in (a–d). The solid magenta line shows the average of the NGC-derived tumour regions; the solid and dashed black lines are the same as in (e).

Brain sections containing NGC-derived tumours were also imaged, at two ROIs, in the same way as described for the sections containing GSC-derived cancerous tissue. In the HIA/FSC^3^ analysis pipeline, the spectral components determined from the ROIs with GSC-derived cancer cells were used as a basis (projected FSC^3^, see section 2.4), allowing for a direct comparison between the two data sets. Concentration errors and spectral errors for this factorisation analysis are shown in Fig. S15–S20† and Fig. S21, S22.† Notably, the spectral errors are similar across the GSC and NGC data sets, showing that both datasets are well represented by the chosen common spectral basis. Colour overlays were generated using the same components mapped in the same RGB order, and are presented in Fig. 7 alongside the colour maps for two ROIs in the GSC-derived tumour sections, on the same colour scale for direct comparison. Firstly, we again note the clear distinction between cancer cells and the normal tissue. Secondly, when comparing the GSC-derived and NGC-derived tumours in the false colour maps we note the GSC-derived cells appear significantly greener. This shows that tumour cells derived from GSCs contain more PNA than those derived from NGCs. A quantitative analysis of the dry concentrations and the water concentration is shown in Fig. 8 confirming this conclusion. Indeed, the spatially-resolved dry PNA concentration *γ*^d^_4_*C*_4_, across all available ROIs, revealed a mean value of 14.6% vol/vol (with a standard deviation (SD) of 0.9%) for the GSC-derived tumour regions compared to a mean of 9% vol/vol (SD 0.5%) in the NGC-derived tumour regions (corresponding to a 1.6 ratio), and a mean of 7.8% vol/vol (SD 0.6%) in the normal tissue regions (see section S2 iii†). Therefore, our quantitative hyperspectral CARS technique not only distinguishes between cancerous *versus* normal brain tissue, but is also able to dissect the heterogeneity of cancer cells, and to distinguish tumours derived from stem cells *versus* non-stem cells.

## 4. Conclusions

In conclusion, we have investigated two types of complex multicellular systems, namely liver organoids and brain tissue sections, using correlative TPF and hyperspectral CARS microscopy combined with our recently developed quantitative and unsupervised data analysis pipeline.

Organoids were maintained alive under hyperspectral CARS measurements, owing to carefully designed culture and imaging conditions. They contained a fluorescence construct (Fucci2aR) which enabled us to selectively tag cells in the S/G2/M phase *via* the fluorescent protein mVenus in the nucleus. Quantitative analysis of the volumetric TPF datasets showed a correlation between increasing fluorescence intensity and nucleus volume, which we used to separate the early S stage from the late S/G2 and G2/M stages. Hyperspectral CARS microscopy measurements acquired on a sub-set of the *z*-planes imaged by TPF provided chemical components, representing water, proteins, DNA, and lipids, with susceptibility spectra and concentration maps in physically meaningful units. An analysis of the average nucleus concentration of the dry components rich in DNA showed a correlation with the trend observed in TPF *versus* nucleus size. The results suggest that label-free chemically specific hyperspectral CARS microscopy can highlight the cell subgroup in the S/G2 phase, when DNA replication has occurred while the cell nucleus size has not yet significantly expanded, such that the DNA concentration is highest.

Brain tissues were sectioned from mice that had been implanted with eGFP-labelled glioblastoma stem cells and non-stem cells. They were imaged with correlative TPF and hyperspectral CARS, the latter analysed to extract meaningful chemical components, namely water, proteins and nucleic acids, lipids, and their concentration maps. Here, we found a correlation between the TPF image contrast from regions containing eGFP-tagged cancer cells and the CARS chemical contrast from regions rich in proteins, clearly distinct from areas containing normal tissue. Remarkably, we were also able to distinguish cancer cells derived from implanted GSCs *versus* NGCs, on the basis of the relative protein content. Specifically we found that tumour cells derived from GSCs are significantly richer in proteins and nucleic acids (by a factor of 1.6 in mean PNA concentration value) compared to those derived from NGCs.

Overall, these results highlight the potential of quantitative hyperspectral CARS microscopy to become an enabling technology in biomedical research, capable of dissecting the complexity of living organs and tissues *via* the label-free identification of chemically distinct subcellular populations non-invasively, offering significant opportunities in *e.g.* 3D cancer biology.

## Data availability

Information about the data created during this research, including how to access it, is available from Cardiff University data archive at http://doi.org/10.17035/d.2020.0123251197.

## Author contributions

T.D., F.S., P.B., W.L. conceptualized the work and methodology. I.P., P.B. and W.L. developed the microscope setup. I.P. acquired the data. F.M. and W.L. developed the analysis software. I.P. and F.M. analysed the data. K.E. prepared and handled the organoids. A.J. and F.S. prepared, stained, and handled the brain slices. I.P. P.B. and W.L. wrote the manuscript. F.M., F.S., and T.D. reviewed the manuscript.

## Conflicts of interest

There are no conflicts to declare.

## Supplementary Material

AN-146-D0AN02381G-s001
