# Supplementary material for: Identifying subpopulations in multicellular systems by quantitative chemical imaging using label-free hyperspectral CARS microscopy
Source: Analyst. 2021 Feb 12;146(7):2277–91. doi: 10.1039/d0an02381g (PMC8359792; doi:10.1039/d0an02381g)
Supplement: AN-146-D0AN02381G-s001 [file AN-146-D0AN02381G-s001.pdf]

Electronic Supplementary Material (ESI) for Analyst.
This journal is © The Royal Society of Chemistry 2021

Supplementary Information - Identifying subpopulations in multicellular systems by quantitative chemical imaging using label-free hyperspectral CARS microscopy

Iestyn Pope,[1] Francesco Masia,[1] Kenneth Ewan,[1] Ana Jimenez-Pascual,[2] Trevor Dale,[1] Florian A. Siebzehnrubl,[2] Paola Borri,[1,*] and Wolfgang Langbein[3,†]

[1] *Cardiff University, School of Biosciences, Museum Avenue, Cardiff CF10 3AX, UK*

[2] *Cardiff University, School of Biosciences, European Cancer Stem Cell Research Institute, Hadyn Ellis Building, Maindy Rd, Cardiff CF24 4HQ, United Kingdom*

[3] *Cardiff University, School of Physics and Astronomy, The Parade, Cardiff CF24 3AA, UK*

CONTENTS

S1. Fucci2aR Liver Bile Duct Organoids 2
i. TPF *z*-stack 2
ii. Calculating nucleus volumes 2
iii. FSC^3 factorisation 5
iv. Calculating the dry volume fraction 9
v. Calculating nucleus area 11
vi. Analysis of non-fluorescent cells 14

S2. Brain tissue with GBM 15
i. GSC-derived tumours - susceptibilities and concentration maps 15
ii. NGC-derived tumours - susceptibilities and concentration maps 21
iii. Dry Spectra and integration ranges 23

References 26

* borrip@cf.ac.uk

† langbeinww@cf.ac.uk

S1. FUCCI2AR LIVER BILE DUCT ORGANOIDS

i. TPF z-stack

TPF images and hyperspectral CARS z-stacks were acquired at 1 µm and 5 µm intervals, respectively, over a z-range of 30 µm. The pixel size in the xy plane was $(86\,\mathrm{nm})^2$. A pixel dwell time of 100 µs and 1 µs and PMT gains of 10^6 and $10^{4.6}$ were used for TPF and CARS, respectively. A montage of all the TPF xy planes is presented in Fig. S1, yellow boxes indicate the z planes where corresponding hyperspectral CARS data sets were acquired. A total of 13 nuclei were identified, and numbered as indicated in Fig. S1.

ii. Calculating nucleus volumes

The *3D Objects Counter* [1] plugin for ImageJ was used to quantitatively analyse the TPF z-stack to determine the nuclei volumes. A filters size of 500 voxels (minimum number of voxels needed for an object to be counted) and an intensity threshold value equivalent to 1.34×10^5 phe/s were used. This individually segmented the nuclei of cells 3, 4, 5, 6, 10, 11 and 12, while nuclei 7, 8, 9, and 13 were joined and required a manual separation as follows. The area around the nucleus was manually traced in all z-planes and all other cells removed, leaving only the nucleus of interest. This was then processed through the *3D Objects Counter* with the same intensity threshold value of 1.34×10^5 phe/s. The TPF signal from the nuclei of cells 1 and 2 is too close to the background and could not be reliably segmented by *3D Objects Counter*, therefore, cells 1 and 2 are not included in any of the subsequent analysis. An overview of the nuclei contour as a result of this segmentation is shown in Fig. S2. For each of the remaining 11 nuclei, the intensity (in phe/s) was summed over all the voxels within the nucleus, and multiplied by the voxel volume (in µm^3), to obtain the integrated intensity. This is plotted against the nucleus volume in the main manuscript Fig.3. Similarly, the integrated intensity divided by the nucleus volume is plotted against nucleus volume.

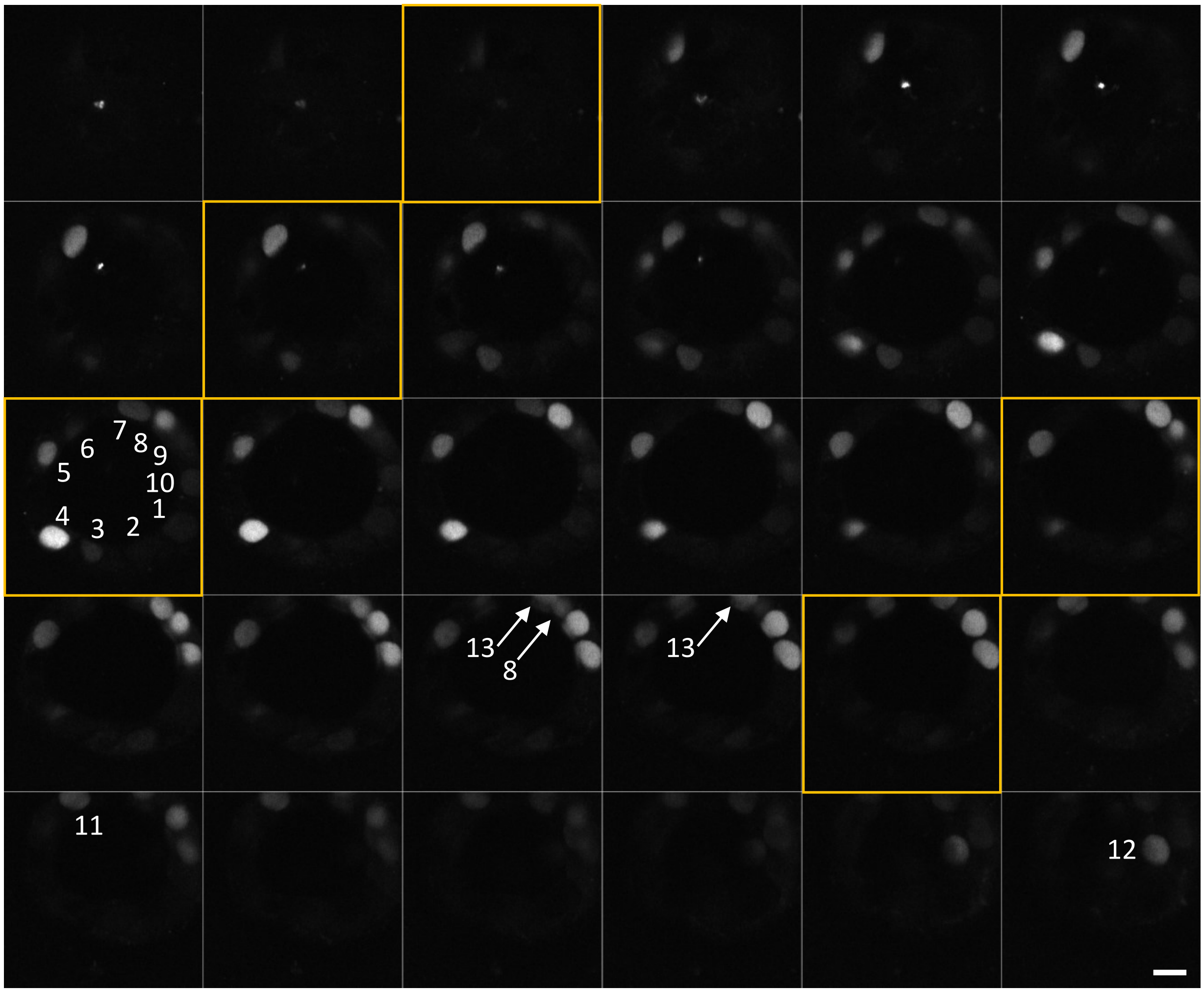

FIG. S1. Montage of all xy planes from the organoid TPF z-stack with 1 µm steps in z, increasing from 0 µm (top left) to the right and down up to 30 µm (bottom right). Yellow box indicate the z planes corresponding to the hyperspectral CARS acquisitions taken at z = 2, 7, 12, 17, and 22 µm. Cells are numbered for the analysis. Images are shown on a grey scale from 0 to 1.1×10^6 photoelectrons per second. Scale bar 10 µm.

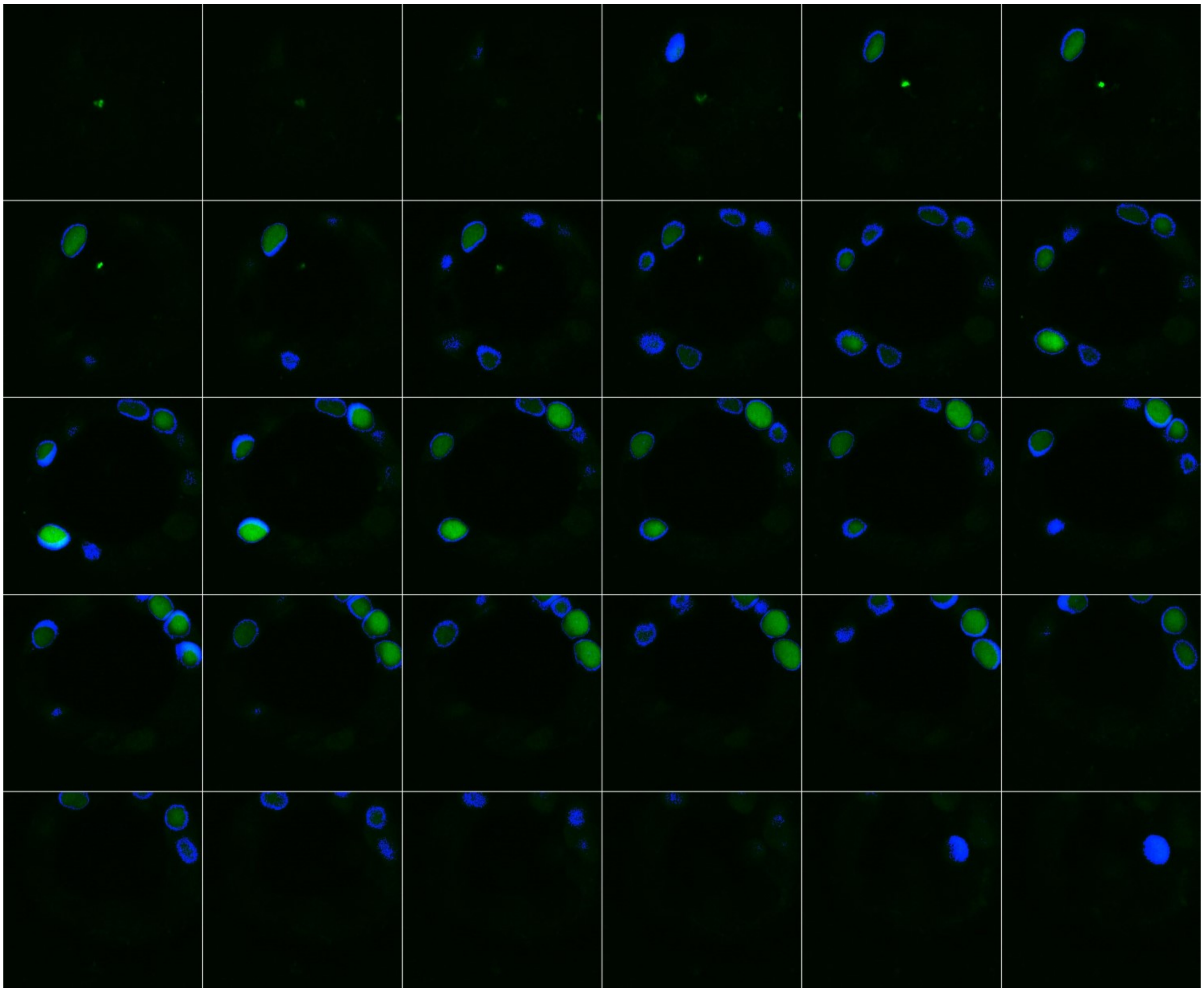

FIG. S2. Montage of all xy planes from the TPF z-stack (same as in Fig.S1), with the TPF intensity in green, superimposed with the nuclei contour voxels obtained from the 3D segmentation in blue.

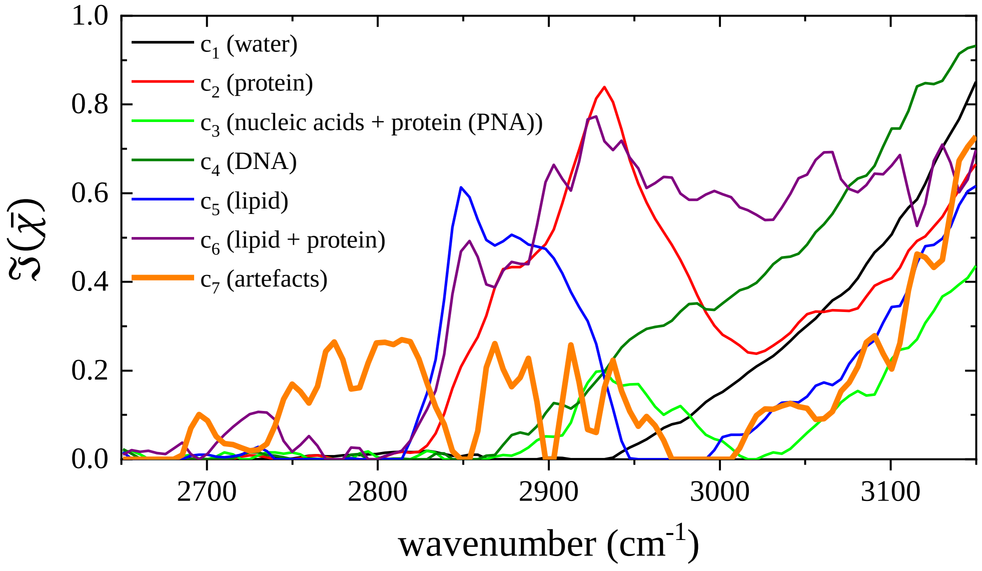

FIG. S3. Susceptibility spectra from the FSC^3 analysis on Fucci2aR liver bile duct organoids.

iii. FSC^3 factorisation

The hyperspectral CARS dataset for the xy plane $z = 12$ µm was processed through our HIA/FSC^3 pipeline. A total of seven components were used for the factorisation which was carried out over the spectral range 2650–3150cm^{-1}. The susceptibility spectra for the seven components determined by the FSC^3 factorisation are shown in Fig. S3. These seven components were then used as the basis for a projected FSC^3 factorisation of the hyperspectral CARS datasets at the other four planes. Component concentration maps, concentration error maps and spectral error maps for each of the five xy planes are given in Figs. S4–S8.

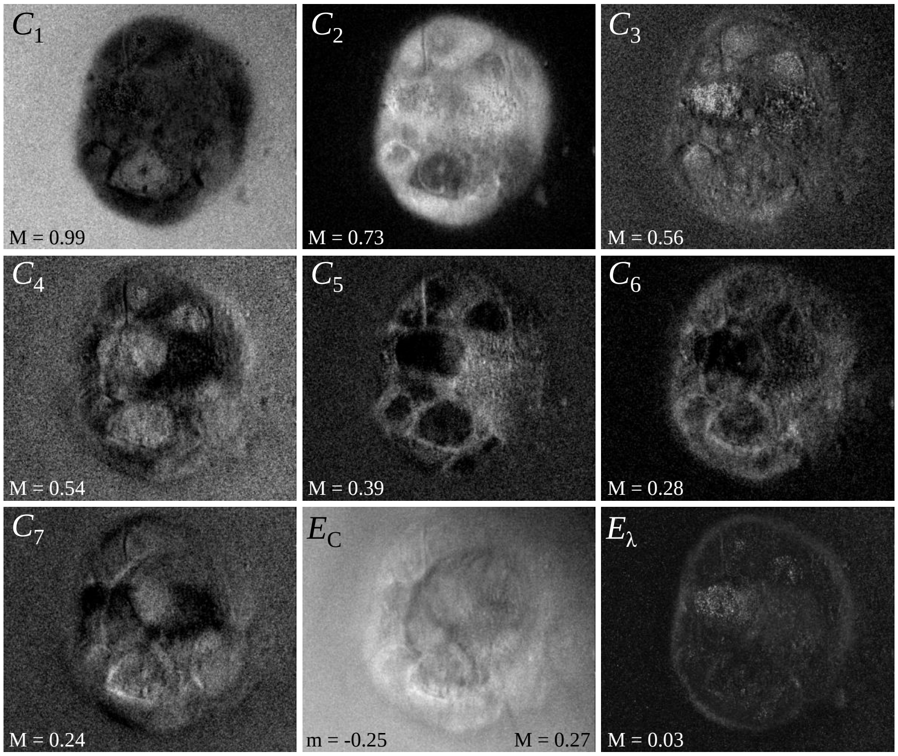

FIG. S4. Concentration and error maps for the xy plane at $z = 2\,\mu$m. Images C_1 to C_7 are the concentration maps of the corresponding susceptibility spectra (see Fig. S3), on a grey scale from 0 to M as given in vol/vol units. E_{C}, concentration error and E_λ spectral error, grey scales from m to M and 0 to M, respectively.

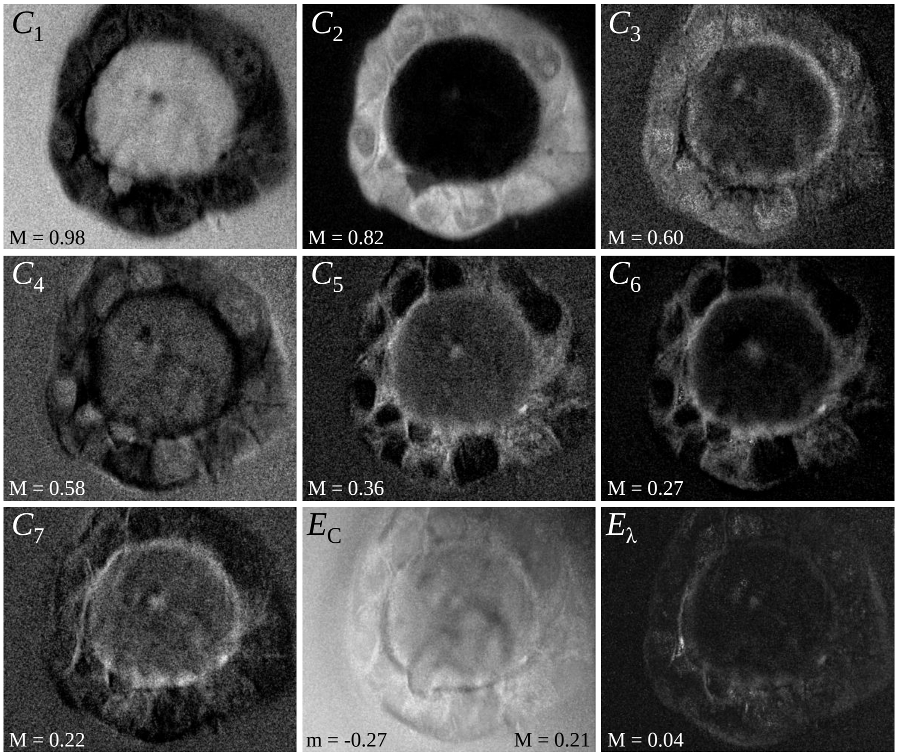

FIG. S5. As Fig. S4, but at $z = 7\,\mu\text{m}$.

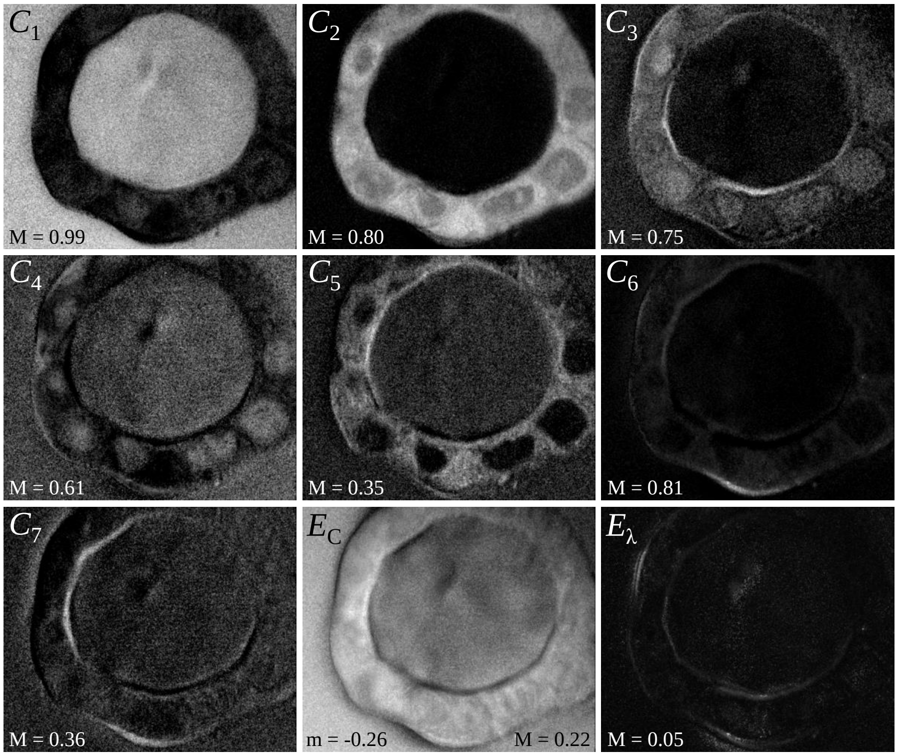

FIG. S6. As Fig. S4, but at $z = 12\,\mu\text{m}$.

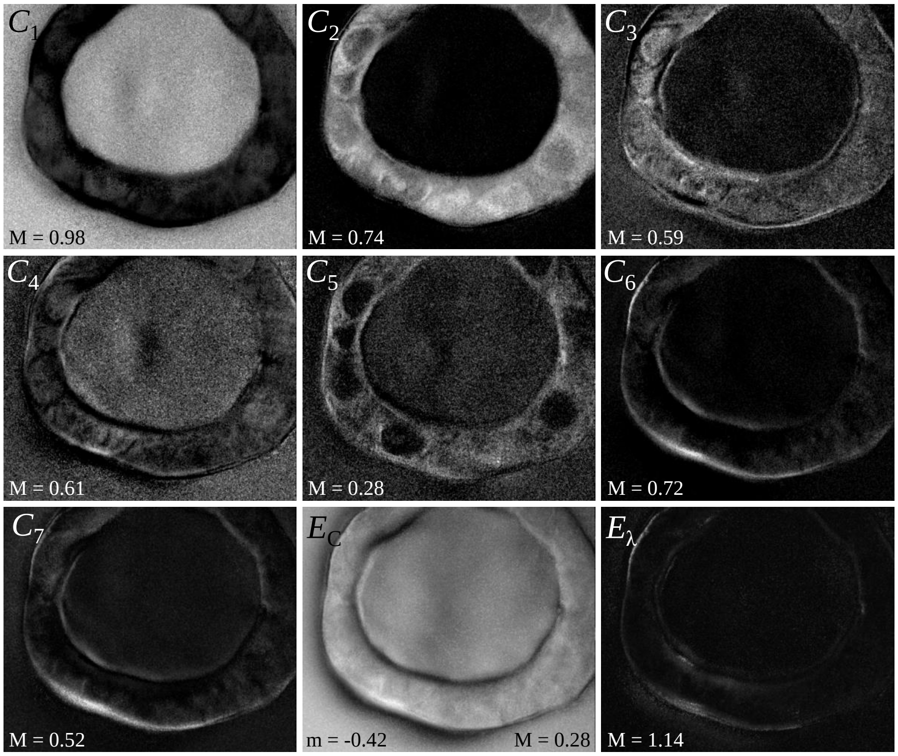

FIG. S7. As Fig. S4, but at $z = 17\,\mu\text{m}$.

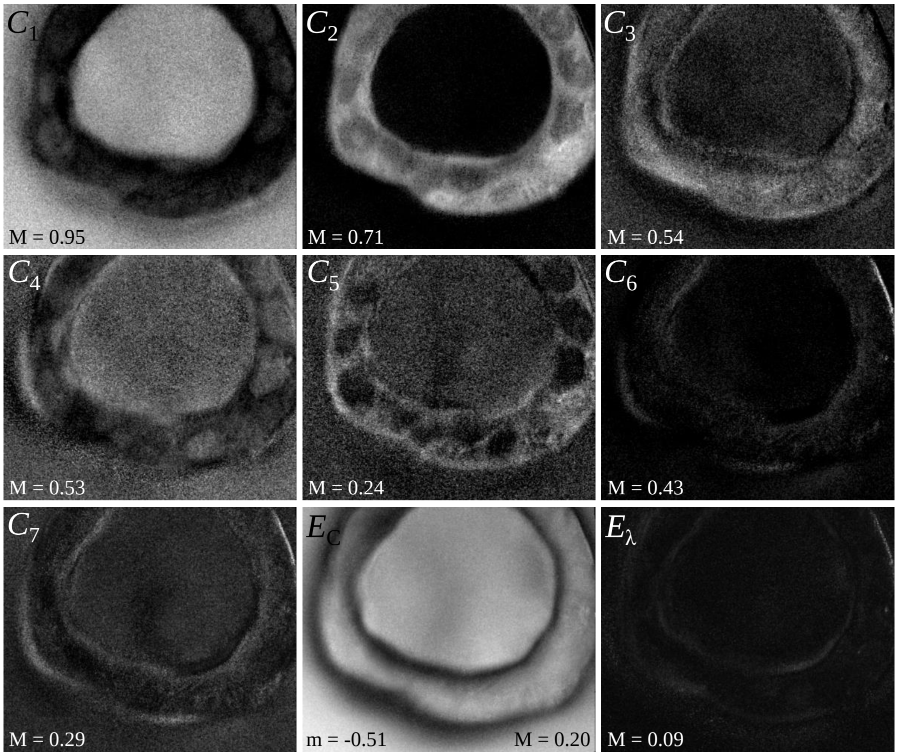

FIG. S8. As Fig. S4, but at $z = 22\,\mu\text{m}$.

iv. Calculating the dry volume fraction

The FSC^3 components often contain a fraction of water, a result of the water being present across the imaged volume in each voxel. This is seen as a rising $\Im(\bar{\chi})$ above $3100\,\mathrm{cm}^{-1}$ in the component spectra. To determine the dry volume fraction of the components, the water part is subtracted from the component spectra and the resulting dry spectra are integrated and compared with known integrals for the dry components. In detail, we subtracting a fraction (α) of the water spectrum $\Im(\bar{\chi})_{\mathrm{W}}$ (component c_1, Fig. S3) from spectrum of the component in question, resulting in its dry spectrum $\Im(\bar{\chi})_{\mathrm{dry}} = \Im(\bar{\chi}) - \alpha\Im(\bar{\chi})_{\mathrm{W}}$ where the fraction α is chosen such that the $\Im(\bar{\chi})_{\mathrm{dry}}$ is zero at $3150\,\mathrm{cm}^{-1}$, as expected for the organic materials investigated.

Following the method outlined by Karuna *et.al.* [2], the dry fraction of component c_i is defined as $\gamma_i^{\mathrm{d}} = A_i/A_{\mathrm{Bulk}}$, where A_i is the spectral integral of $\Im(\bar{\chi})_{\mathrm{dry}}$ for component c_i, and A_{Bulk} is the corresponding integral of the dry bulk material of the component. The latter is estimated as $A_{\mathrm{Bulk}} = A_{\mathrm{OA}}F_i$, where A_{OA} is the spectral integral of the susceptibility measured for a known pure dry material (we used oleic acid, OA) and the factor F_i is calculated assuming that the integral is proportional to the volume density of -H bonds. The factor F_i can be expressed as

$$F_{\mathrm{i}} = \frac{b_i}{b_{\mathrm{OA}}}\frac{m_{\mathrm{OA}}}{m_i}\frac{\rho_i}{\rho_{\mathrm{OA}}} \tag{1}$$

where b_{OA} (b_i) is the number of Raman active bonds in a molecule of OA (c_i) in the wavenumber range considered, m_{OA} (m_i) is the molecular weight of OA (c_i) and ρ_{OA} (ρ_i) is the mass density of OA (c_i), respectively. The values used to calculate A_{Bulk} are taken from Karuna *et.al.* [2] and are given in table S1 alongside the integrals calculated from the dry spectra of S9. The dry vol/vol concentration is then taken as the dry fraction multiplied by the component concentration ($\gamma_i^{\mathrm{d}} C_i$).

Comp	Attribution	A_i (cm^{-1})	b_i	m_i (amu)	ρ_i (g/cm^3)	F_i	$A_{\mathrm{OA}}F_i$	γ_i^{d}
c_2	PNA (mostly protein)	83.2	6	128.44	1.3	0.581	462.9	0.1797
c_3	PNA	19.6	-	-	1.5	0.564	449.3	0.0437
c_4	DNA	39.8	18	650	2	0.530	422.2	0.0942
c_5	lipid (OA)	56.6	33	282.47	0.895	1	797.0	0.0710
c_6	PNA+lipid (mostly protein)	127.1	6	128.44	1.3	0.581	462.9	0.2745
c_7	artefacts	38.8	-	-	-	-	-	-

TABLE S1. Parameters used in the calculation of dry fractions γ_i^{d}. Values for A_i were calculated from the dry spectra shown in Fig. S9, all other values were taken from Karuna *et.al.* [2]. $b_{\mathrm{OA}} = 33$, $\rho_{\mathrm{OA}} = 0.895$ g/cm^3, $m_{\mathrm{OA}} = 282.47$ amu, $A_{\mathrm{OA}} = 797$ cm^{-1}.

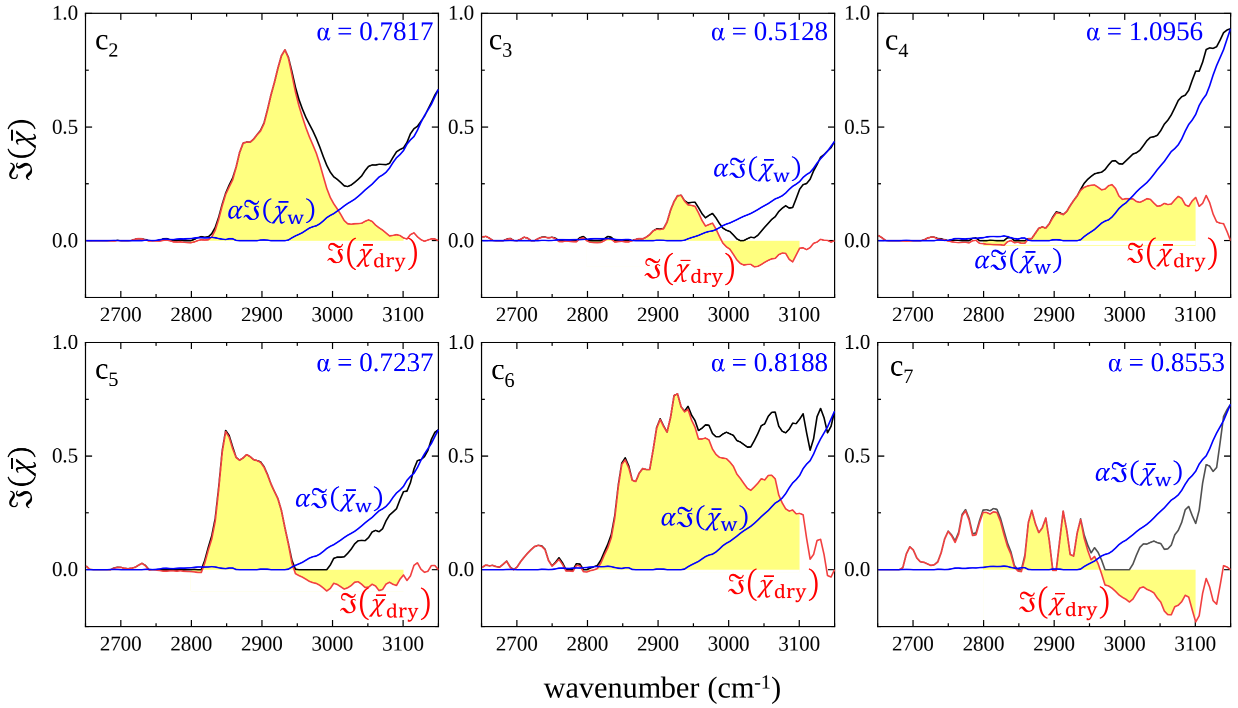

FIG. S9. Dry fraction estimation for components c_2 to c_7 shown in Fig. S3. From the $\Im(\bar{\chi})$ of c_i (black), the scaled water spectrum $\alpha\Im(\bar{\chi})_{\mathrm{W}}$ (blue) is subtracted, resulting in $\Im(\bar{\chi})_{\mathrm{dry}}$ (red) for each component. Areas of integration over the range 2800 cm^{-1} to 3100 cm^{-1} are shown in yellow; corresponding integrals are given in table S1.

v. Calculating nucleus area

The concentration map of component c_5 clearly shows the nucleus area, see Fig. S4–S8. For each xy plane imaged by CARS, the nucleus region was traced by hand in ImageJ. These regions were then used as templates to calculate $\gamma_i^{\mathrm{d}} C_i$ averaged over the nucleus area, for the concentration maps of components $i = 2$ to 6. TPF images were used to identify nuclei positive for the mVenus marker, and were labelled according to the assignment given in Fig. S1. Cell 1 and 2 were excluded, as they had a too low TPF intensity to reliable segment a nucleus volume, as explained in section S1 ii. If a nucleus appeared in more than one z plane, it was assigned to the same number in all z planes. Cells 11, 12 and 13 were not captured in any of the hyperspectral CARS planes and are thus not included. For the dry component analysis, only those nuclei where the CARS plane was near the nucleus centre were included. This was done to avoid artefacts by the objective focal volume extending beyond the nuclei volume. Overlays of the nuclei regions and numbered assignments are given in Fig. S10, where numbers are shown in green for nuclei that were used in the analysis, and in white if they were not used. Corresponding plots for each component averaged over the nucleus area, from $\gamma_2^{\mathrm{d}} C_2$ to $\gamma_6^{\mathrm{d}} C_6$, as a function of the nucleus volume, are shown in Fig. S11.

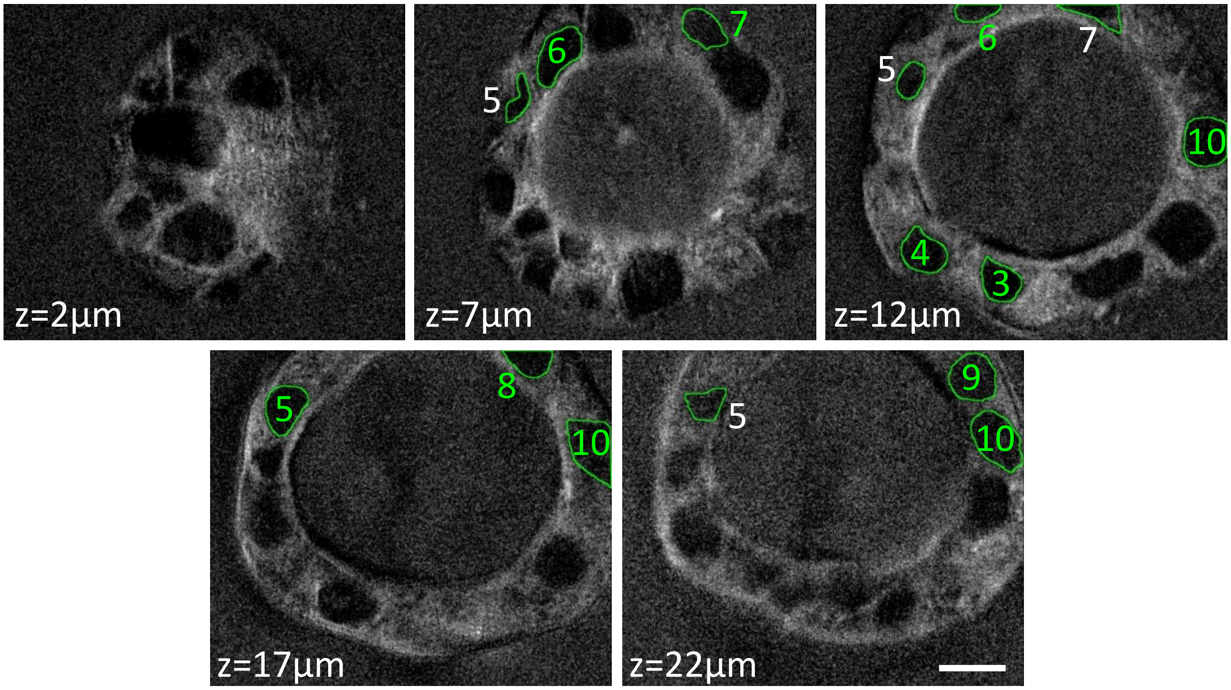

FIG. S10. Concentration maps for component c_5 showing the defined nuclei regions used to calculate $\gamma_i^{\mathrm{d}} C_i$. Numbers in green (white) indicate nuclei that were used (not used) in the analysis, respectively. Scale bar 10 µm.

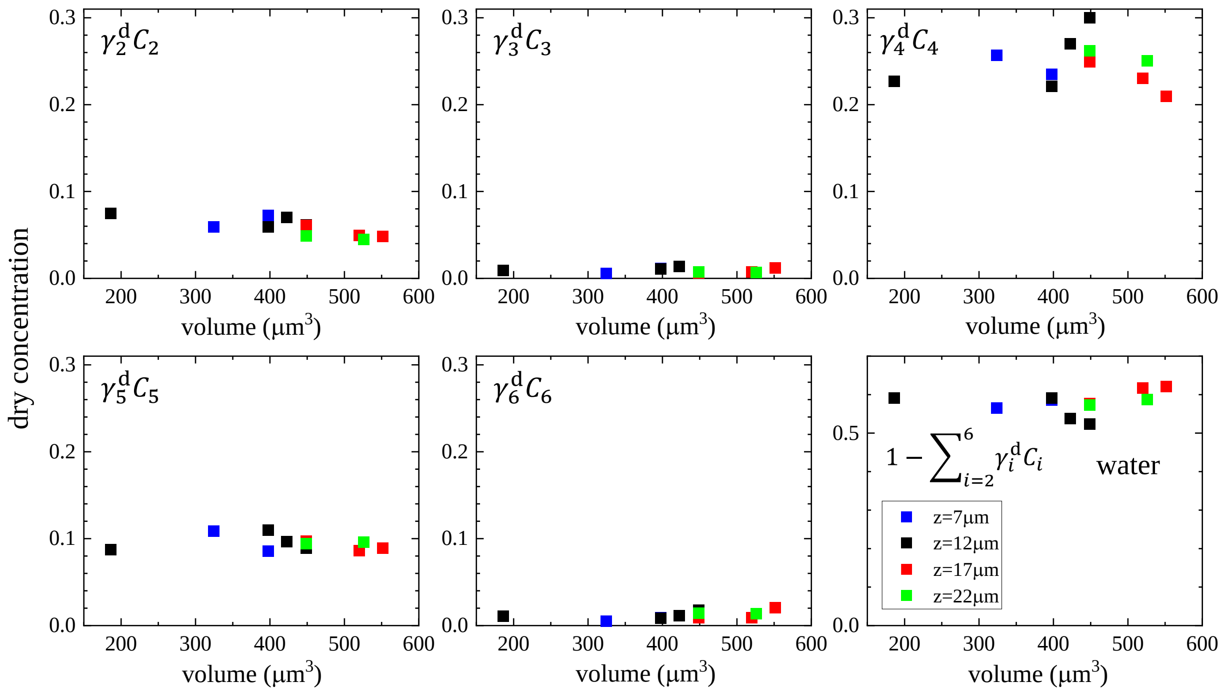

FIG. S11. Dry concentration of components averaged over the nuclei areas, for the cells that exhibited mVenus TPF, as a function of the nucleus volume (each data point represents an individual nucleus). Different z-planes were evaluated (indicated by colours), using a common basis of components, and data are shown for those planes where the nucleus was largest (see text).

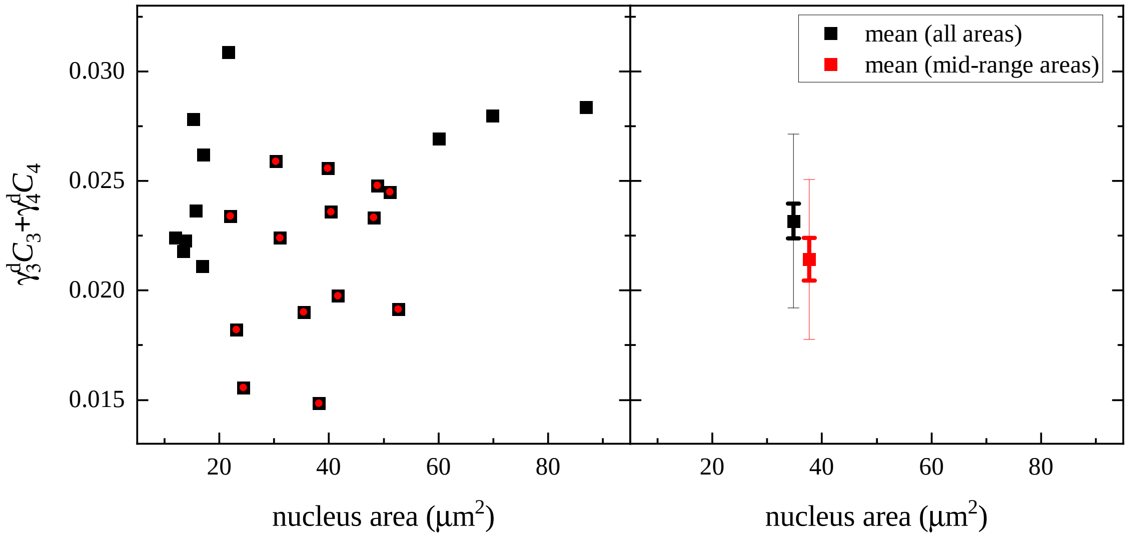

FIG. S12. Left: Dry concentration component $\gamma_3^{\mathrm{d}} C_3 + \gamma_4^{\mathrm{d}} C_4$ averaged over the nucleus areas, for all cells observed in CARS microscopy that did not exhibit detectable mVenus TPF in the nucleus, as a function of the nucleus area (each data point represents an individual nucleus). The sub-set of cells having a nucleus area from $22\,\mu\mathrm{m}^2$ to $53\,\mu\mathrm{m}^2$ are shown as red data points. Right: mean (symbol), standard deviation (thin bar) and standard error of the mean (thick bar) for the data points shown on the left.

vi. Analysis of non-fluorescent cells

The dry concentration $\gamma_3^{\mathrm{d}} C_3 + \gamma_4^{\mathrm{d}} C_4$ averaged over the nucleus area was also calculated for all cells that were imaged by hyperspectral CARS and did not exhibit a mVenus TPF localization in the nucleus. Nuclei areas were segmented in the hyperspectral CARS images, using the same procedure discussed in section S1 v. We considered two cases: all the nuclei areas, and a sub-set whereby we excluded nuclei with small areas ($< 22\,\mu\mathrm{m}^2$), which could be too close to the top/bottom edges. In this sub-set, we also excluded nuclei with very large areas ($> 53\,\mu\mathrm{m}^2$), to select cells in the G1 phase. An overview of $\gamma_3^{\mathrm{d}} C_3 + \gamma_4^{\mathrm{d}} C_4$ for each cell in the two sets, and the resulting mean, standard deviation, and standard error of the mean is shown in Fig. S12.

S2. BRAIN TISSUE WITH GBM

i. GSC-derived tumours - susceptibilities and concentration maps

For the mouse brain tissues xenografted with eGFP-labelled GBM stem cells (GSC), six regions of interest (ROIs) containing both cancer and normal cells were initially found, using wide-field epi-fluorescence and DIC imaging. A z-position approximately in the centre of the 30 μm-thick section was located using TPF imaging (Fig. S13). Alongside the TPF images, hyperspectral CARS was acquired over the range 2600–3700 cm^{-1}, with 5 cm^{-1} step size. Hyperspectral CARS was processed using our HIA/FSC^3 pipeline. FSC^3 analysis was carried out over the spectral range 2700–3100 cm^{-1}. Susceptibility spectra of all five components are given in Fig. S14, with the component concentration maps, concentration error maps and spectral error maps for each ROI shown in Fig. S15–S20. RGB overlays were generate from the concentration maps C_2 (red), C_4 (green) and $C_1 + C_3$ (blue) for each of the six ROIs, and are shown in Fig. S13 rows 2 and 4.

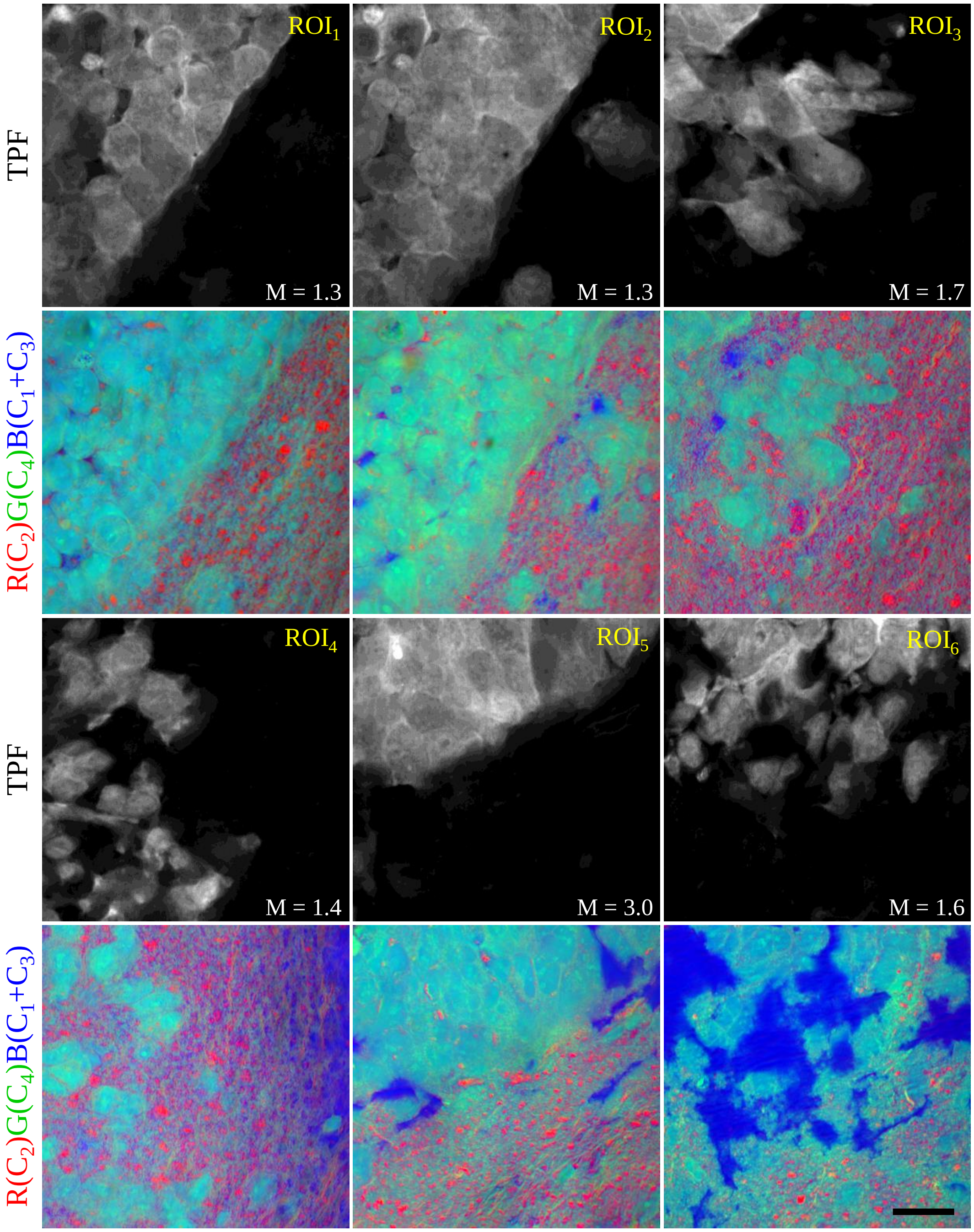

FIG. S13. Overview of all six ROIs for the samples with GSC-derived tumour cells. Rows 1 and 3 are TPF images, grey scale range 0 to M$\times 10^8$ photoelectrons per second (see inset in image for M values). ROIs are labelled from left to right, as ROI_1 to ROI_3 in row 1 and ROI_4 to ROI_6 in row 3. Rows 2 and 4 are false-colour maps generated from the overlay of concentration maps (Figs. S15 to S20) C_2 (red), C_4 (green), $C_1 + C_3$ (blue), on the same RGB scale, from 0 to a colour maximum scale M (vol/vol) with $\mathrm{M}_{\mathrm{red}} = 0.78$, $\mathrm{M}_{\mathrm{green}} = 0.32$, $\mathrm{M}_{\mathrm{blue}} = 0.84$. Scale bar: 20 µm.

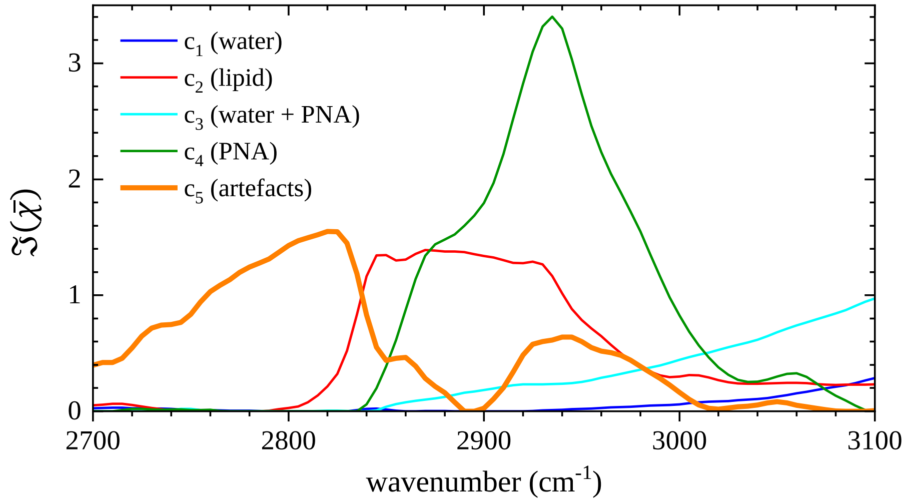

FIG. S14. Susceptibility spectra for the FSC^3 analysis on samples with GSC-derived tumours. All six ROIs were analysed together over the range 2700–3100 cm^{-1}.

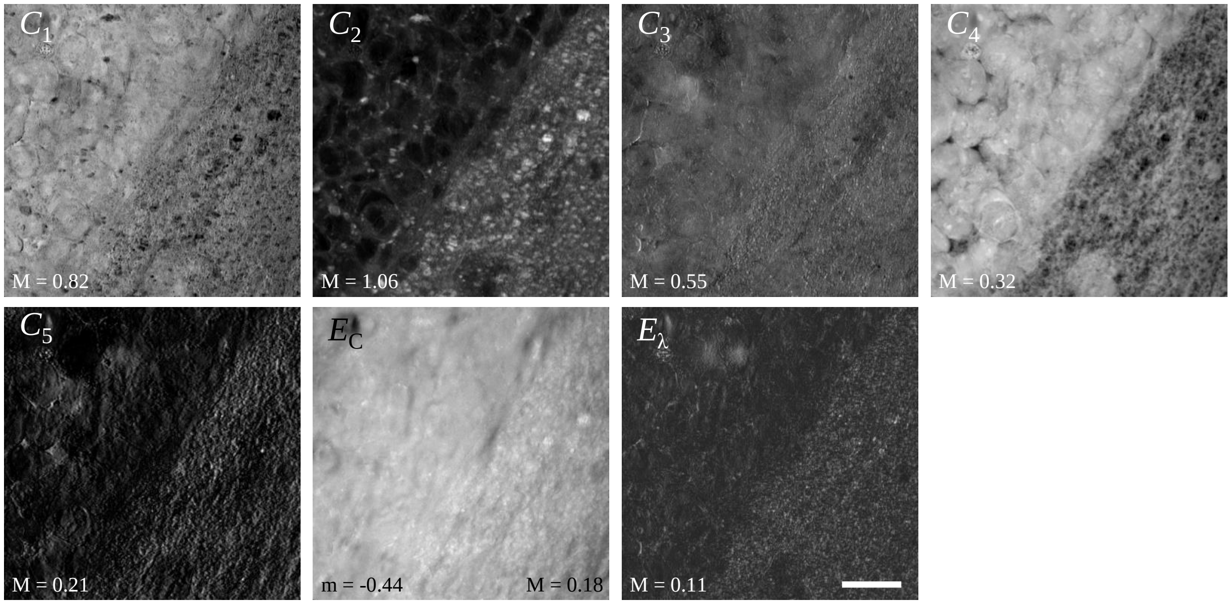

FIG. S15. Overview of concentration maps for GSC sample ROI$_1$. Images C_1 to C_5 are the concentration maps of the corresponding susceptibility spectra (Fig.S14), in grey scale from 0 to M (see inset in image for value) in vol/vol units. E_C, concentration error and E_λ spectral error of the FSC^3 factorisation, grey scales from m to M and 0 to M respectively, as given. Scale bar 20 µm.

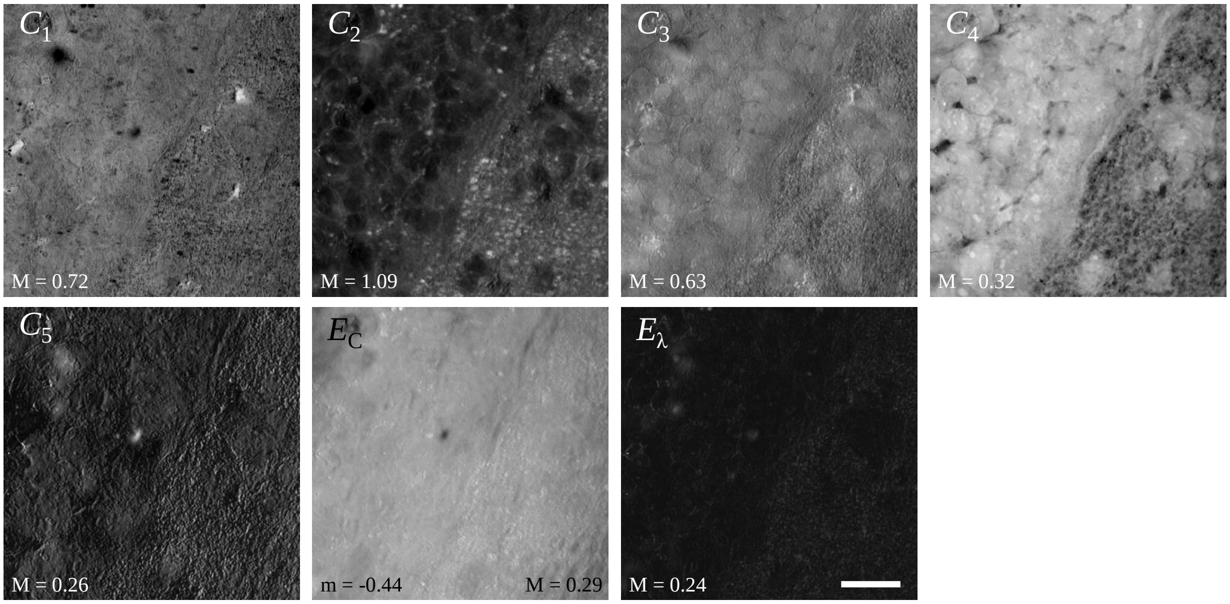

FIG. S16. As Fig. S15 but for ROI_2.

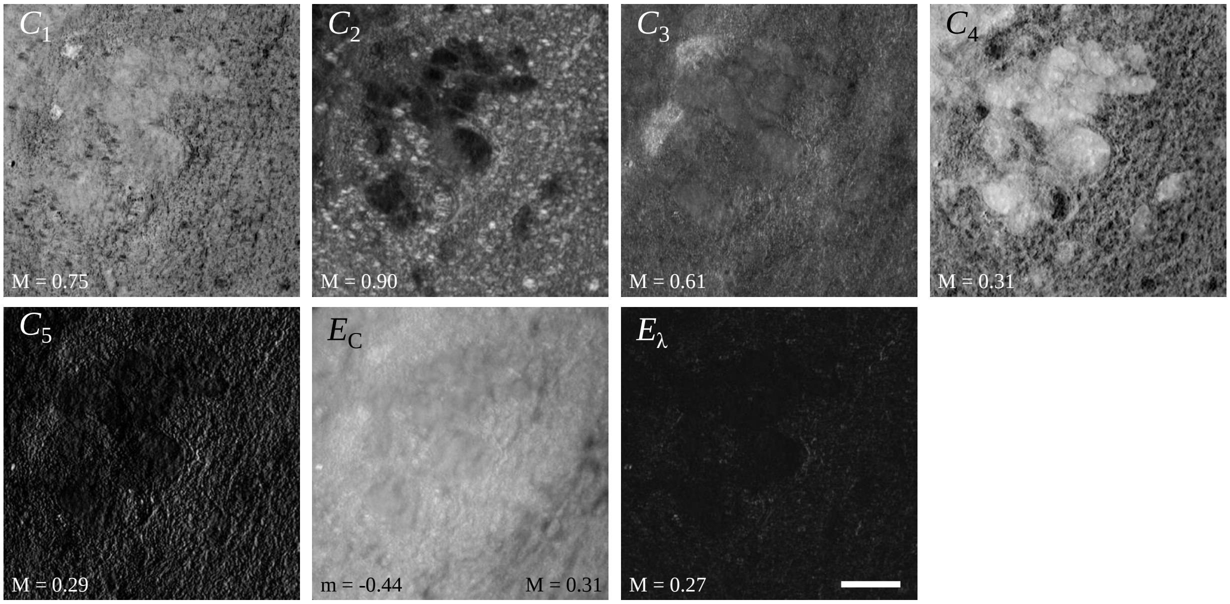

FIG. S17. As Fig. S15 but for ROI_3.

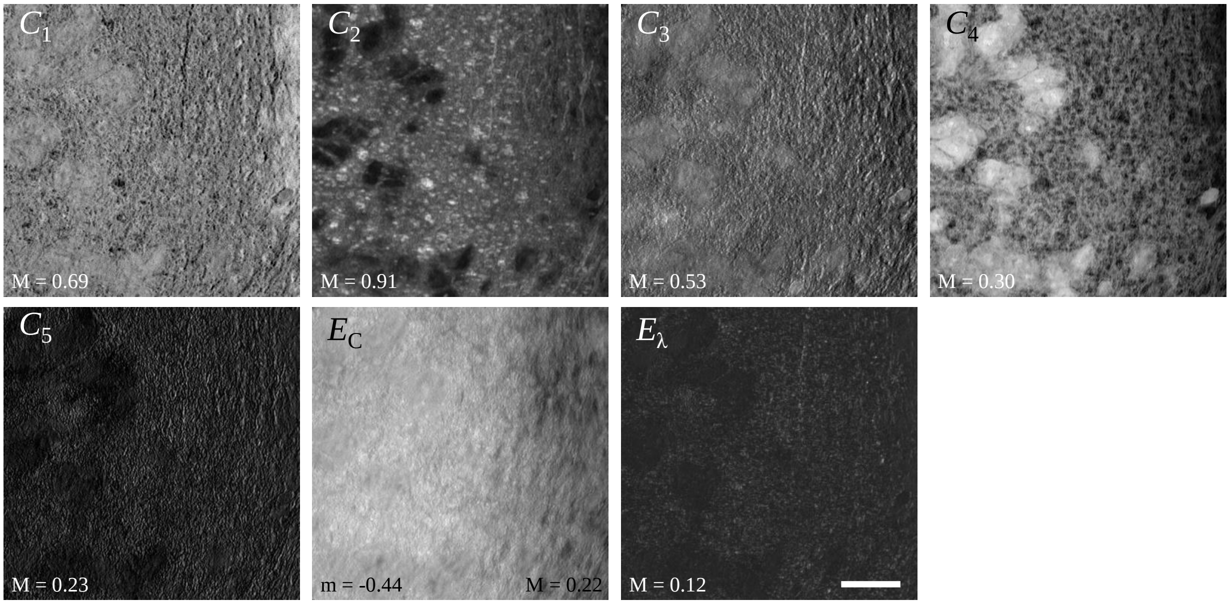

FIG. S18. As Fig. S15 but for ROI_4.

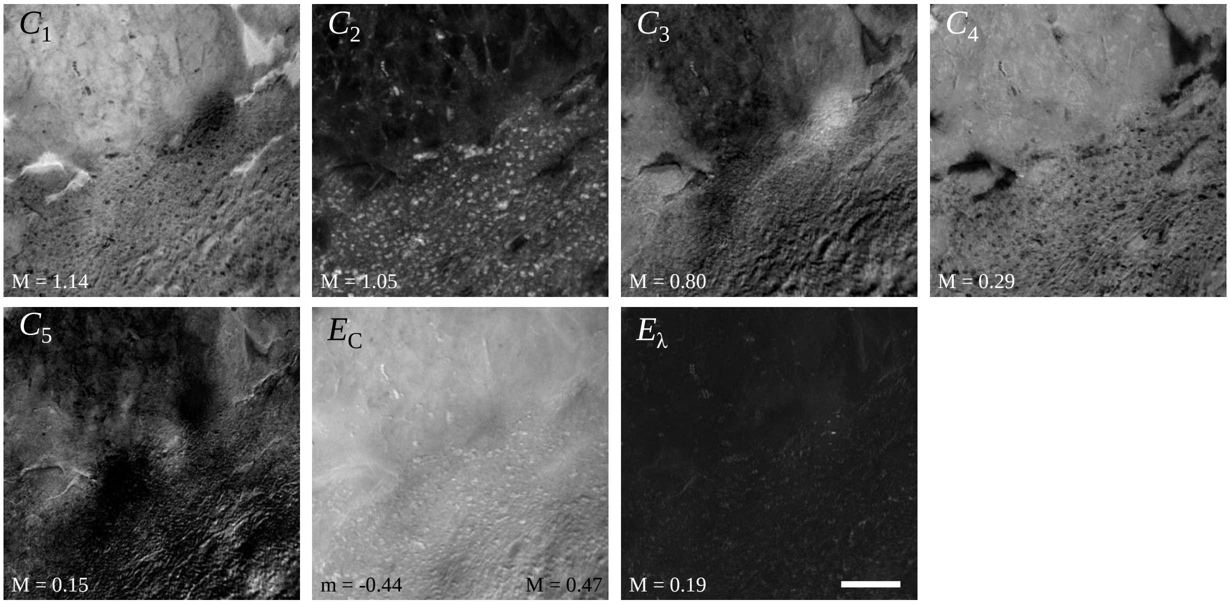

FIG. S19. As Fig. S15 but for ROI_5.

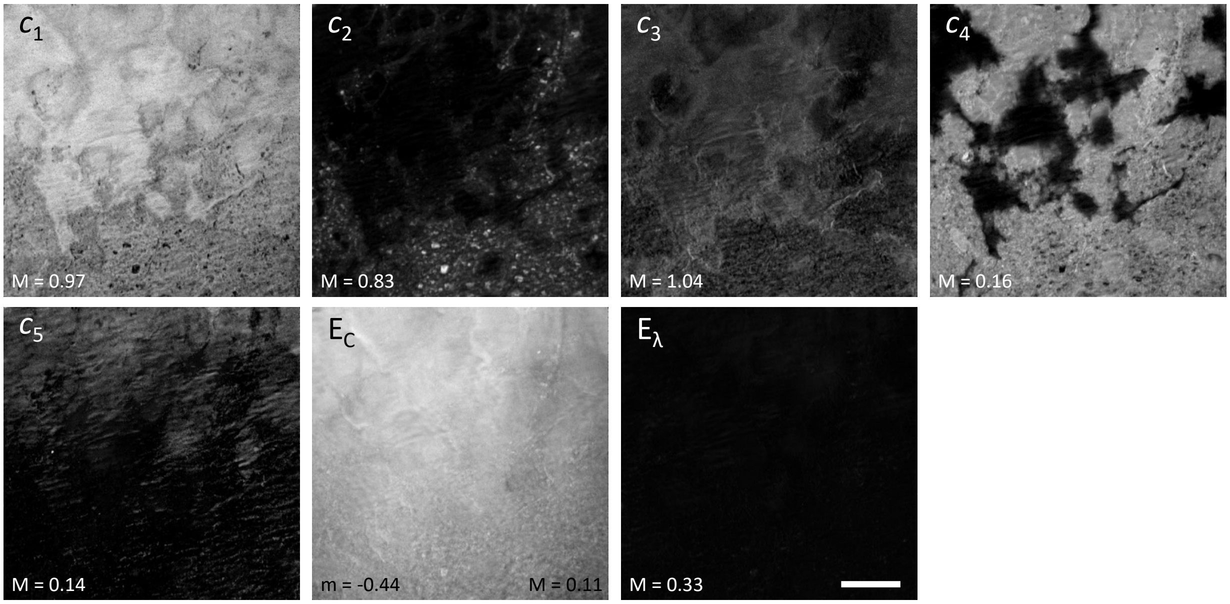

FIG. S20. As Fig. S15 but for ROI_6.

ii. NGC-derived tumours - susceptibilities and concentration maps

Brain tissue sections obtained from mice xenografted with eGFP-labelled non-stem GBM cells (NGC) cells were imaged, at two ROIs, in the same way as described for the tissue sections from mice xenografted with eGFP-labelled GSC cells. In the HIA/FSC^3 analysis pipeline, the spectral components determined from the six ROIs with GSC-derived cancer cells were used as a basis (projected FSC^3), allowing for a direct comparison between the two data sets. The component concentration maps, concentration error maps and spectral error maps for the two ROIs for these NGC samples are given in Figs. S21 and S22.

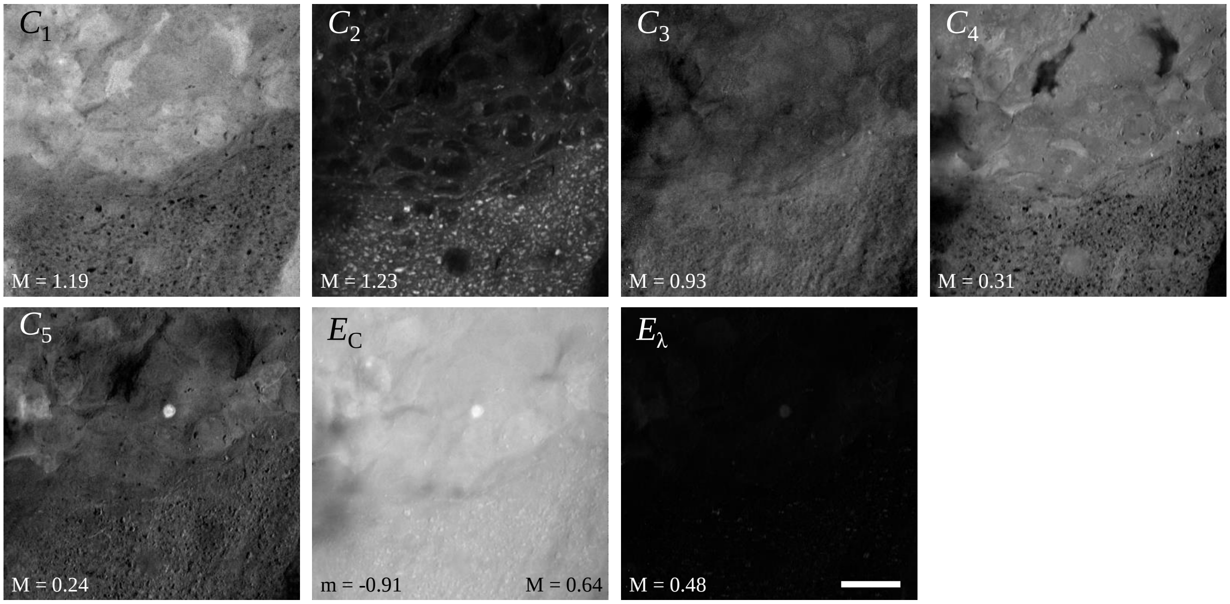

FIG. S21. As Fig. S15 but for NGC sample ROI$_1$.

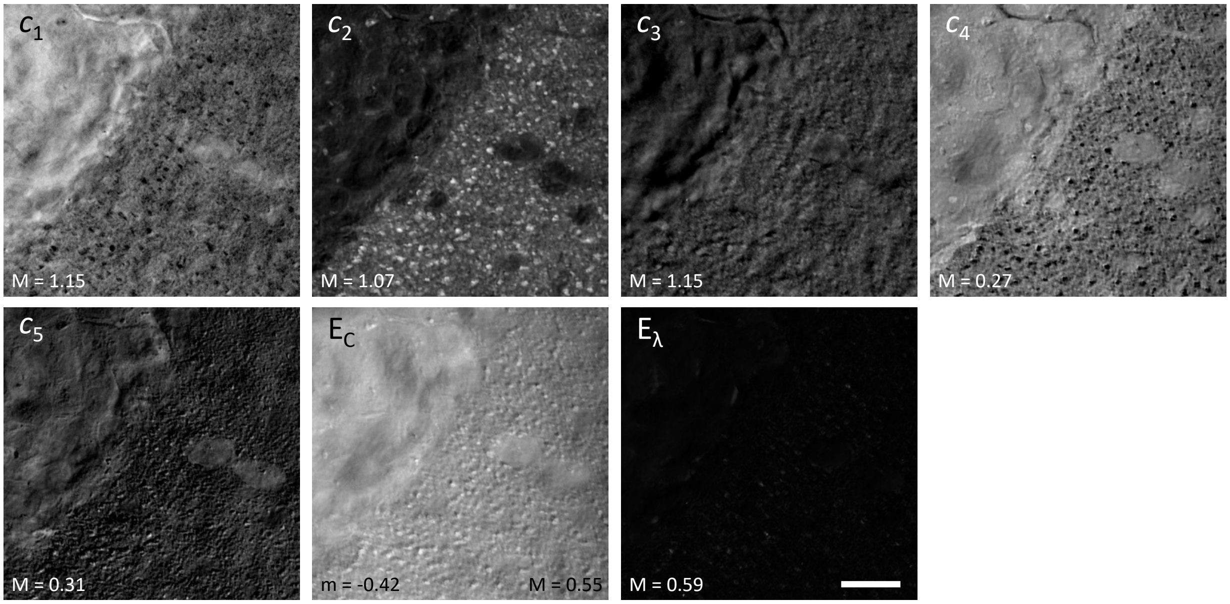

FIG. S22. As Fig. S15 but for NGC sample ROI_2.

Comp	Attribution	A_i (cm^{-1})	b_i	m_i (amu)	ρ_i (g/cm^3)	F_i	$A_{\mathrm{OA}}F_i$	γ_i^{d}
c_2	lipid (OA)	188.6	33	282.47	0.895	1	797.0	0.2367
c_3	PNA	46.4	6	128.44	1.3	0.581	462.9	0.1003
c_4	water + PNA	321.0	6	128.44	1.3	0.510	462.9	0.6935
c_5	artefacts	121.2	-	-	-	-	-	-

TABLE S2. Parameters used in the calculation of dry fractions γ_i^{d}. Values for A_i were calculated from the dry spectra shown in Fig. S23, all other values were taken from Karuna *et.al.* [2]. $b_{\mathrm{OA}} = 33$, $\rho_{\mathrm{OA}} = 0.895$ g/cm^3, $m_{\mathrm{OA}} = 282.47$ amu, $A_{\mathrm{OA}} = 797$ cm^{-1}.

iii. Dry Spectra and integration ranges

Dry spectra were calculated as described in section S1 iv. The dry spectra for components c_2 to c_5 are shown in Fig. S23. Fig.S24 and S25 show the overlays used to mark out regions containing either tumour cells (regions marked out in black) or normal tissue (regions marked out in white). These regions were then used to calculate $\gamma_4^{\mathrm{d}} C_4$ as average concentrations, for both the GSC and NGC samples. See table S2 for γ_i^{d} values.

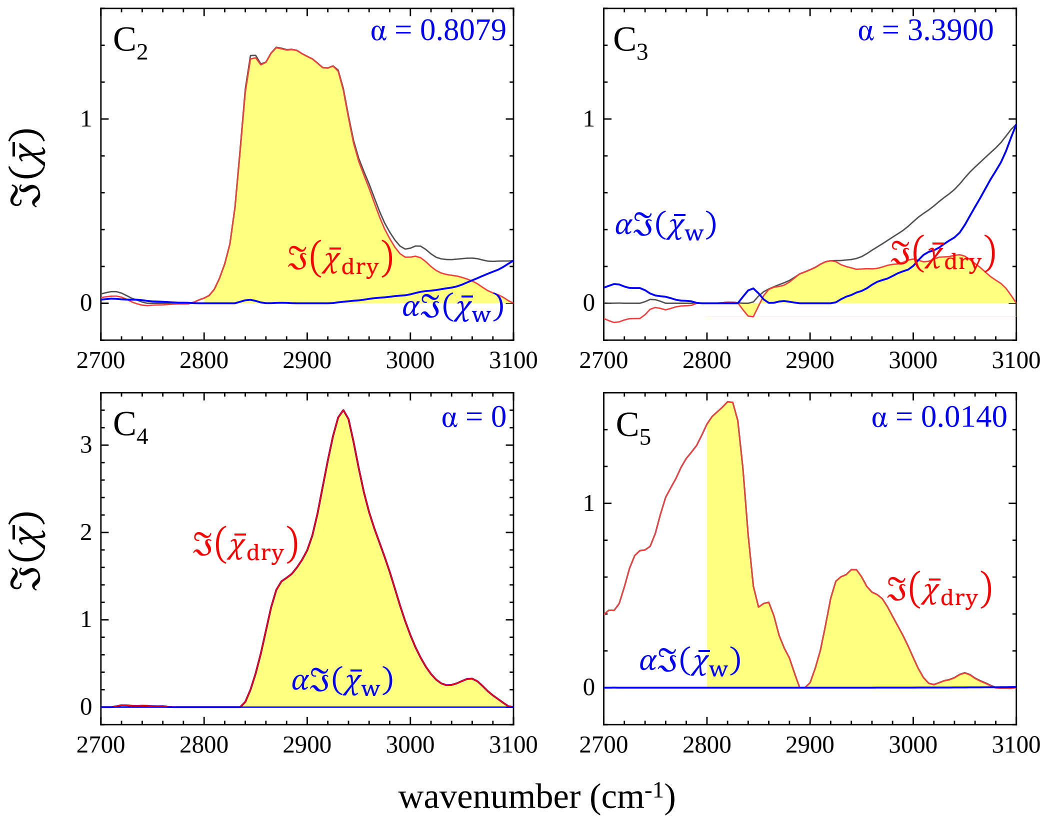

FIG. S23. Dry fraction estimation for component c_2 to c_5 shown in Fig. S14. From the $\Im(\bar{\chi})$ of c_i (black), the scaled water spectrum $\alpha\Im(\bar{\chi})_{\mathrm{W}}$ (blue) is subtracted, resulting in $\Im(\bar{\chi})_{\mathrm{dry}}$ (red) for each component. Areas of integration over the range 2800 cm^{-1} to 3100 cm^{-1} are shown in yellow, corresponding integrals are given in table S2.

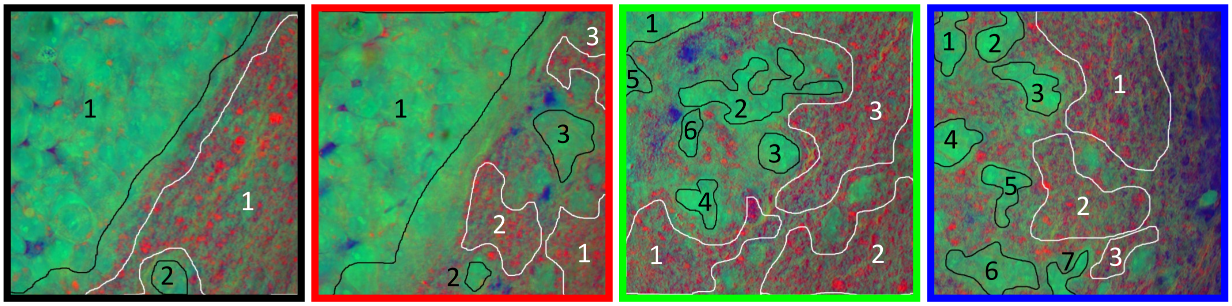

FIG. S24. False colour RGB images from Fig. 6 showing the overlays used to mark out regions containing either tumour cells (regions marked out in black) or normal tissue (regions marked out in white). Coloured frames indicate the colour-coding used for the data points in Fig. S26 (graph, left).

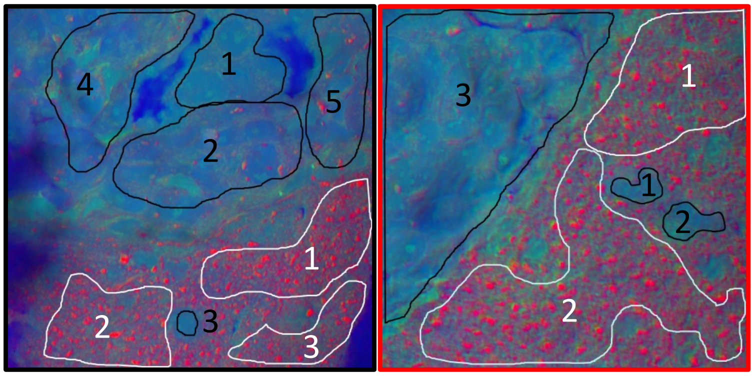

FIG. S25. False colour RGB images on NGC samples from Fig. 7 showing the overlays used to mark out regions containing either tumour cells (regions marked out in black) or normal tissue (regions marked out in white). Coloured frames indicate the colour-coding used for the data points in Fig. S26 (graph, right).

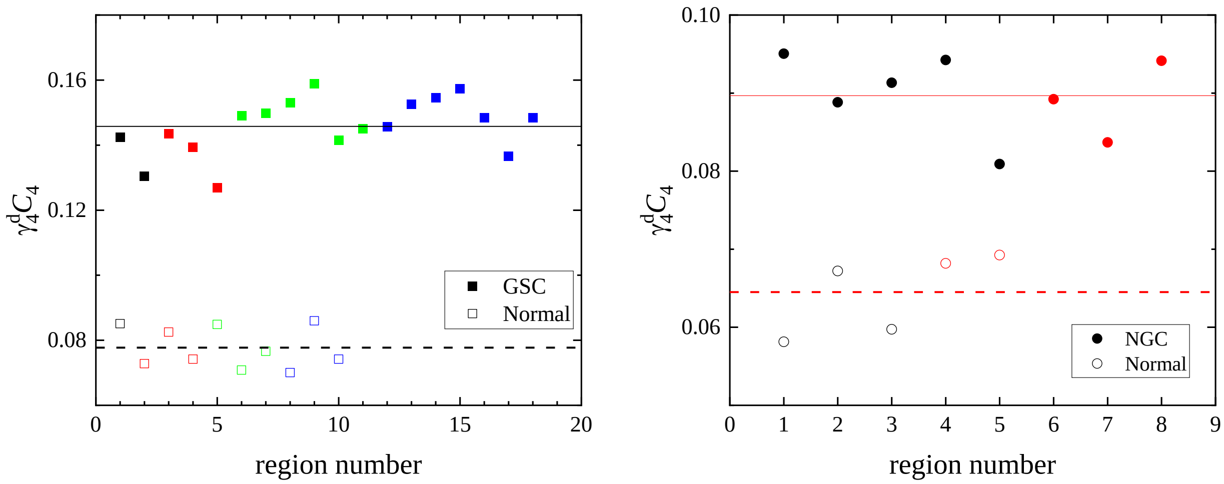

FIG. S26. Protein component dry concentration $\gamma_4^{\rm d}C_4$ (vol/vol) spatially averaged over selected regions identified by Fig. S24 and S25. Left: GSC samples $\gamma_4^{\rm d}C_4$ (vol/vol) for the regions indicated Fig. S24. Right: NGC samples, $\gamma_4^{\rm d}C_4$ (vol/vol) for the regions indicated Fig. S25. Data points are colour-coded according to the frame colours around the images. Solid lines give average value across tumour regions. Dashed lines give average value for normal tissue regions. These graphs are the same data shown in the main paper in Fig. 7, with the addition here of region colour-coding for clarity.

[1] Bolte, S. & Cordèlieres, F. P. A guided tour into subcellular colocalization analysis in light microscopy. *J. Microsc.* **224**, 213–232 (2006).

[2] Karuna, A. *et al.* Label-Free Volumetric Quantitative Imaging of the Human Somatic Cell Division by Hyperspectral Coherent Anti-Stokes Raman Scattering. *Anal. Chem.* **91**, 2813–2821 (2019).
